# Water–fat Dixon cardiac magnetic resonance fingerprinting

**DOI:** 10.1002/mrm.28070

**Published:** 2019-11-18

**Authors:** Olivier Jaubert, Gastão Cruz, Aurélien Bustin, Torben Schneider, Begoña Lavin, Peter Koken, Reza Hajhosseiny, Mariya Doneva, Daniel Rueckert, René M. Botnar, Claudia Prieto

**Affiliations:** ^1^ School of Biomedical Engineering and Imaging Sciences King’s College London London United Kingdom; ^2^ Philips Healthcare Guilford United Kingdom; ^3^ Philips Research Europe Hamburg Germany; ^4^ Department of Computing Imperial College London London United Kingdom; ^5^ Escuela de Ingeniería Pontificia Universidad Católica de Chile Santiago Chile

**Keywords:** cardiac MRI, fat fraction, MR fingerprinting, T_1_ mapping, T_2_ mapping, water–fat DIXON

## Abstract

**Purpose:**

Cardiac magnetic resonance fingerprinting (cMRF) has been recently introduced to simultaneously provide T_1_, T_2_, and M_0_ maps. Here, we develop a 3‐point Dixon‐cMRF approach to enable simultaneous water specific T_1_, T_2_, and M_0_ mapping of the heart and fat fraction (FF) estimation in a single breath‐hold scan.

**Methods:**

Dixon‐cMRF is achieved by combining cMRF with several innovations that were previously introduced for other applications, including a 3‐echo GRE acquisition with golden angle radial readout and a high‐dimensional low‐rank tensor constrained reconstruction to recover the highly undersampled time series images for each echo. Water–fat separation of the Dixon‐cMRF time series is performed to allow for water‐ and fat‐specific T_1_, T_2_, and M_0_ estimation, whereas FF estimation is extracted from the M_0_ maps. Dixon‐cMRF was evaluated in a standardized T_1_–T_2_ phantom, in a water–fat phantom, and in healthy subjects in comparison to current clinical standards: MOLLI, SASHA, T_2_‐GRASE, and 6‐point Dixon proton density FF (PDFF) mapping.

**Results:**

Dixon‐cMRF water T_1_ and T_2_ maps showed good agreement with reference T_1_ and T_2_ mapping techniques (R^2^ > 0.99 and maximum normalized RMSE ~5%) in a standardized phantom. Good agreement was also observed between Dixon‐cMRF FF and reference PDFF (R^2^ > 0.99) and between Dixon‐cMRF water T_1_ and T_2_ and water selective T_1_ and T_2_ maps (R^2^ > 0.99) in a water–fat phantom. In vivo Dixon‐cMRF water T_1_ values were in good agreement with MOLLI and water T_2_ values were slightly underestimated when compared to T_2_‐GRASE. Average myocardium septal T_1_ values were 1129 ± 38 ms, 1026 ± 28 ms, and 1045 ± 32 ms for SASHA, MOLLI, and the proposed water Dixon‐cMRF. Average T_2_ values were 51.7 ± 2.2 ms and 42.8 ± 2.6 ms for T_2_‐GRASE and water Dixon‐cMRF, respectively. Dixon‐cMRF FF maps showed good agreement with in vivo PDFF measurements (R^2^ > 0.98) and average FF in the septum was measured at 1.3%.

**Conclusion:**

The proposed Dixon‐cMRF allows to simultaneously quantify myocardial water T_1_, water T_2_, and FF in a single breath‐hold scan, enabling multi‐parametric T_1_, T_2_, and fat characterization. Moreover, reduced T_1_ and T_2_ quantification bias caused by water–fat partial volume was demonstrated in phantom experiments.

## 1 INTRODUCTION

Quantitative parametric mapping has been increasingly incorporated into clinical cardiovascular MR examinations to provide objective myocardial tissue characterization^1^ of both focal and diffuse diseases, including assessment of fibrosis, inflammation,^2^ and edema.^3^ Several mapping methods, including MOLLI,^4^ SASHA,^5^ T_2_‐prepared bSSFP,^3^ and T_2_‐GRASE,^6^ have been proposed to measure myocardial T_1_ and T_2_ independently. Conventionally, T_1_ and T_2_ mapping acquisitions are performed in several separate 2D scans, at different slice positions, and under breath‐holding, potentially leading to mis‐registration between the different acquisitions^7^, ^8^ and ultimately contributing to considerable patient fatigue (contributing to the several tens of breath‐holds required in a conventional clinical cardiac protocol). Furthermore, these mapping techniques typically rely on simplified exponential relaxation models to estimate T_1_ and T_2_ values and, in general, do not account for system imperfections.

To accelerate parameter mapping in cardiac MR, joint relaxometry techniques have been proposed to estimate T_1_ and T_2_ simultaneously, relying on magnetization preparation with varying T_1_ and T_2_ weightings and combined relaxometry models.^9^, ^10^, ^11^ These methods reduce the total number of breath‐holds and produce co‐registered T_1_ and T_2_ maps. An alternative approach for efficient joint T_1_ and T_2_ tissue mapping is magnetic resonance fingerprinting (MRF).^12^ MRF uses a highly undersampled transient state acquisition scheme with varying acquisition parameters that causes the signals from different tissues to have a unique signal evolution or fingerprint. Matching the measured MR signal response to a previously generated dictionary of fingerprints allows MR tissue identification and parameter estimation. Fingerprints are designed as a function of multiple tissue parameters and therefore several quantitative parameters can be simultaneously reconstructed from the same single acquisition, without the need of relying on simplified relaxation models.

In particular, cardiac MRF (cMRF)^13^, ^14^ has been recently introduced for mapping myocardial T_1_, T_2_, and M_0_ during a single breath‐hold scan. Different to conventional MRF, cMRF acquisition uses variable magnetization preparation with interleaved inversion recovery (IR) and T_2_ preparation (T_2_prep) pulses to increase sensitivity to T_1_ and T_2_ parameters.^13^ Furthermore, the cMRF approach does not acquire data continuously: ECG‐triggering with a short acquisition window (<250 ms) is used to minimize cardiac motion, whereas breath‐holding (<20 s) is needed to minimize respiratory motion, therefore limiting the total effective acquisition time to ~4 s. Additionally, the dictionary of fingerprints needs to be recomputed for each subject to account for heart rate variability. cMRF has shown promising results in comparison to conventional T_1_ and T_2_ mapping techniques and has the potential to be extended to map other clinically relevant parameters.^14^

In addition to T_1_ and T_2_ mapping, fat characterization plays an important role in evaluating cardiovascular disease. Cardiac fat has been shown to carry important diagnostic information to characterize lipomatous metaplasia,^15^ which has high prevalence and prognostic value in patients with myocardial infarction.^16^, ^17^ Moreover, water and fat partial volume is a known source of error in parametric mapping,^18^ and previous works have explored simultaneous T_1_ and fat imaging to reduce partial volume effects and quantify epicardial fat volumes.^19^ Therefore, the extension of cardiac MRF to enable additional fat fraction (FF) quantification and partial volume correction is desired.

Multi‐compartment^20^, ^21^ and water–fat^22^, ^23^, ^24^, ^25^, ^26^ MRF approaches have been recently proposed for static body parts such as knee, liver, breast, upper thigh, and whole‐body. There are several challenges in extending such methods for cardiac applications. For one, these approaches require longer acquisition times, ranging from 10–24 s of continuous acquisition per slice, making them incompatible with the requirements of cardiac imaging, i.e., short effective scan times ~4 s to enable clinically feasible breath‐holding (~15 s). In previous approaches to enable water–fat MRF, additional parameters such as B_0_ and/or B_1_ needed to be included in the dictionary for correction. The correction was achieved in earlier water–fat MRF works using separate acquisitions to obtain the field maps^25^, ^26^ or adding the additional parameters as part of the dictionary matching step.^22^, ^23^, ^24^ Both techniques significantly increase the dictionary size (and corresponding computation time) without providing additional diagnostic information. Since cardiac MRF requires subject‐specific dictionaries, shortening dictionary computation times is essential to maintain feasible reconstruction times.

In order to address the limitations of previously introduced techniques for cardiac imaging, in this work, we propose a 3‐point Dixon cardiac MRF (Dixon‐cMRF) framework to enable simultaneous water‐specific T_1_, T_2_, and M_0_ myocardial mapping and fat fraction (FF) estimation from a single breath‐hold scan, while potentially reducing T_1_ and T_2_ biases caused by water–fat partial volume. This is achieved by synergistically combining and extending previously proposed approaches to satisfy the requirements of cardiac MRF imaging. The proposed Dixon‐cMRF framework integrates (1) a 3‐echo gradient‐echo golden angle radial readout to enable short TRs and reduced fat blurring (as opposed to spiral readouts previously used in cardiac MRF); (2) a B_0_ and B_1_ insensitive cMRF acquisition scheme,^27^ avoiding the acquisition of additional B_1_ and B_0_ maps (that would require additional breath‐holds and would be prone to mis‐registration errors) or adding B_1_ and B_0_ as parameters in the dictionary (that would highly increase the dictionary size and computational time); (3) a patch‐based multi‐contrast low‐rank tensor reconstruction^28^ to recover the highly undersampled time series images for each echo, satisfying scan times constraints; and (4) a chemical shift‐based method^29^ to separate the water and fat time series in the compressed temporal domain before independent water and fat MRF matching, avoiding the generation of large dictionaries. The proposed Dixon‐cMRF framework was evaluated in a standardized T_1_/T_2_ phantom, a water–fat phantom, and healthy subjects. In vivo results were qualitatively and quantitatively compared to conventional SASHA, MOLLI, and T_2_‐GRASE mapping in 10 subjects and 6‐point Dixon proton density fat fraction (PDFF) in 3 of these subjects.

## 2 METHODS

### 2.1 Acquisition

**Figure 1:**
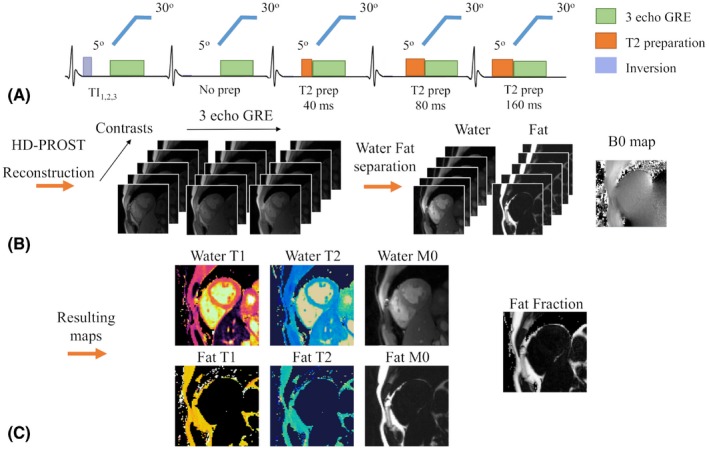
Proposed Dixon‐cMRF framework. (A) A 3‐echo gradient echo golden radial acquisition is ECG‐triggered to acquire k‐space data at the mid‐diastolic cardiac phase for 15 heartbeats (i.e., ~15 s). Magnetization preparation includes inversion pulses every 5 heartbeats (with inversion delays of 10, 300, and 10 ms, respectively) and variable T_2_ preparation modules (no T_2_ preparation, no T_2_ preparation, 40, 80, and 160 ms repeated over 15 heartbeats). (B) The 3 echoes are reconstructed as separate time series from which a B_0_ map estimate and water–fat separated time series are obtained. (C) After a matching step, water‐ and fat‐specific T_1_, T_2_, and M_0_ maps and fat fraction can be extracted

The proposed Dixon‐cMRF consists of a 15‐heartbeat ECG‐triggered golden angle radial (~111°) gradient rewound echo (GRE) acquisition with varying inversion and T_2_ preparation pulses (Figure 1). Inversion pulses are applied every 5 heartbeats with delays of [10, 300, 10] ms, whereas T_2_ preparations with TEs of [No T_2_prep, No T_2_prep, 40, 80, 160] ms are repeated 3 times over the 15 heartbeats. Additional contrast encoding is provided with the flip angle pattern composed of a linear ramp‐up of 20 RF pulses from 5° to 30° followed by a fixed 30° flip angle.^27^ Low flip angles, fixed TR and gradient spoiling were used to reduce the signal’s sensitivity to B_0_ and B_1_ inhomogeneities.^27^, ^30^ Excitations were performed using a short asymmetrical sinc pulse with a time bandwidth product of 3. Three echoes are sampled using bipolar gradients within each TR.

### 2.2 Image reconstruction

HD‐PROST,^28^ a recently introduced multi‐contrast, patch‐based, high‐order, low‐rank reconstruction, is used to highly accelerate the proposed Dixon‐cMRF acquisitions while still providing high quality maps. Dictionary‐based global temporal compression of the MRF time‐series^31^, ^32^ is exploited through singular value decomposition (SVD), to reduce undersampling artefacts in the images. This so‐called low rank inversion (LRI) approach^32^ replaces the reconstruction of the whole MRF time‐series xʹ by the reconstruction of a reduced number R of singular images. These singular images are a low rank approximation of the MRF time‐series and are obtained by projecting the time series onto a subspace identified through SVD of the dictionary. They are defined as $x=U_{R}^{H}x^{\prime }$ where the columns of **U** *_R_* are the truncated (to rank R) left singular vectors of the dictionary matrix **D**. LRI reconstruction is formulated as the following optimization problem

(1) $$\hat{x}={\text{argmin}}_{x}\frac{1}{2}{∥{\mathrm{AU}}_{R}\mathrm{FCx}-k∥}_{2}^{2},$$

where **F** is the Fourier transform operator, **k** is the undersampled k‐space data, **A** is the sampling operator, and **C** are the coil sensitivity maps. The singular images **x** estimated with this method can still present remaining undersampling artefacts and lead to noisy parametric maps. HD‐PROST^28^ reconstruction proposes to further exploit local (within a patch), non‐local (between patches in a neighborhood), and spectral (between contrasts) redundancies through high‐order low‐rank regularization.^33^, ^34^ HD‐PROST reconstructs the multi‐contrast Dixon‐cMRF singular images **x** *_i_* for each echo *i*, by jointly solving

(2) $$L\left({\hat{x}}_{i},\;{\hat{T}}_{b}\right):={\text{argmin}}_{x_{i},T_{b}}\frac{1}{2}{∥{\mathrm{AU}}_{R}{\mathrm{FCx}}_{i}-k∥}_{2}^{2}+\lambda \sum_{b}{∥T_{b}∥}_{*}\;s.t.\;T_{b}=P_{b}\left(x_{i}\right),$$

where ***P*** *_b_*(·) is the operator that assembles a third order tensor $T_{b}$ for the patch centered on voxel *b* by concatenating the K most similar patches along the non‐local similarity dimension (similar patches within a neighborhood), and the R contrasts reconstructed along the spectral dimension (singular images), whereas λ is the corresponding parameter promoting low‐rank regularization.

### 2.3 Water/fat separation

Considering a water (**W′**) and a set of fat ($F_{k}^{\prime }$) compartments time‐series,^35^, ^36^ the reconstructed singular images at echo *i*, can be written as

(3) $$\begin{array}{cc} x_{i}\; & =U_{R}^{H}x_{i}^{\prime }=U_{R}^{H}\left(W^{\prime }+\sum_{k}F_{k}^{\prime }e^{j2\pi \Delta f_{k}t_{i}}\right)e^{j2\pi \Delta f_{B0}\left(t_{i}-t_{1}\right)}\\ & =\left(W+\sum_{k}U_{R}^{H}F_{k}^{\prime }e^{j2\pi \Delta f_{k}t_{i}}\right)e^{j2\pi \Delta f_{B0}\left(t_{i}-t_{1}\right)}=\left(W+F\right)e^{j2\pi \Delta f_{B0}\left(t_{i}-t_{1}\right)}, \end{array}$$

where **W** and $F=U_{R}^{H}\sum_{k}F_{k}^{\prime }e^{j2\pi \Delta f_{k}t_{i}}$ are the temporally compressed water and fat (or combined fat compartments) time series, Δ*f_k_* is the difference in precession frequency between water and fat compartment k, Δ*f_B_*_0_ is the precession frequency difference induced by B_0_ field inhomogeneities, and *t_i_* is the echo time *i*.

The temporally compressed signal formulation in Equation 3 leads to the same water–fat separation problem as for conventional (non‐MRF) multi‐echo Dixon acquisitions.^35^, ^36^ Therefore, the MRF water–fat separation problem can be solved in the compressed temporal domain given an initial B_0_ estimation. A single B_0_ map can be obtained from the first singular images of each echo, which have high SNR ratios. The initial B_0_ estimation is performed using a multi‐seed region growing scheme from 3‐point data and then used for water–fat separation^29^ using a pre‐defined 6‐peak fat model^37^ without accounting for $T_{2}^{*}$ decay.

### 2.4 Feature extraction

The Dixon‐cMRF dictionary was simulated for a range of T_1_/T_2_ values using the extended phase graph (EPG) formalism.^38^ The subject‐specific dictionary was generated using the simultaneously recorded ECG signal, to account for varying acquisition times resulting from intra‐ and inter‐subject heart rate variations. For higher mapping accuracy, the EPG simulation includes a slice profile correction with 51 isochromats^39^ and a Bloch simulation of the inversion efficiency $\delta \left(T_{1},\;T_{2}\right)=\frac{r\left(t+,T_{1},T_{2}\right)}{r\left(t-,T_{1},T_{2}\right)}$, where r is the magnitude of the magnetization vector.

Maps are extracted through dot product matching between the dictionary **D** in the compressed domain and the water–fat separated time series, **W** and **F**, to obtain water–fat‐specific T_1water/fat_, T_2water/fat_, and M_0water/fat_ maps. Dixon‐cMRF FF maps are estimated from the MRF M_0_ maps as^40^

(4) $$\text{FF}=\left\{\begin{array}{cc} \frac{\left|M_{0fat}\right|}{\left|M_{0water}e^{j\varphi_{w}}+M_{0fat}e^{j\varphi_{f}}\right|} & \text{if}\;M_{0fat}>M_{0water}\\ 1-\frac{\left|M_{0water}\right|}{\left|M_{0water}e^{j\varphi_{w}}+M_{0fat}e^{j\varphi_{f}}\right|} & \text{otherwise} \end{array}\right.$$

where $e^{j\varphi_{w}}$ and $e^{j\varphi_{f}}$ are the initial water and fat phase and are estimated for each pixel as the mean phase from the portion of the MRF water and fat signals with positive longitudinal magnetization. Single echo T_1_, T_2_, and M_0_ maps, similar to conventional cMRF, are obtained by matching the 1st, 2nd, or 3rd Dixon‐cMRF reconstructed echo to the same dictionary for comparison purposes.

### 2.5 Experiments

Dixon‐cMRF was evaluated in a standardized T_1_/T_2_ phantom, an in‐house built water–fat phantom, and in vivo in 10 healthy subjects (5 female, age: 31 ± 3.4 y, heart rate: [min, 46; max, 79] beats/min). Data was acquired on a 1.5T MR scanner (Ingenia, Philips Healthcare, the Netherlands) with a 28‐channel cardiac coil. The study was approved by the Institutional Review Board and written informed consent was given by all participants before imaging.

The dictionary included signal evolutions for a range (denoted as [lower value: step size: upper value]) of T_1_s of [50:10:1400, 1430:30:1600, 1700:100:2200, 2400:200:3000] ms and a range of T_2_s of [5:2:80, 85:5:150, 160:10:300, 330:30:600] ms as well as the standardized T_1_/T_2_ phantom^41^ reference values.

The echo images were reconstructed using HD‐PROST (Equation 2). The reconstruction parameter values were chosen empirically based on those used in Bustin et al^28^ and are listed in Supporting Information Figure S1. A reduced patch size of 5 × 5 was used here because of the lower resolution of Dixon‐cMRF compared to the MRF scans reported in Bustin et al.^28^ The rank R = 6 was selected based on the decay of the singular values from the dictionary (Supporting Information Figure S2A). The number of ADMM iterations was selected based on the convergence of the algorithm (Supporting Information Figures S2B and C).

Dixon‐cMRF reconstruction was performed in MATLAB (The MathWorks, Natick, MA) and took ~3 h, including 2.5 h for dictionary generation, 27 min for HD‐PROST reconstruction, 0.3 s for water–fat separation, and 3.2 s for matching of the water and fat time series on a Linux workstation with 8 Intel Xeon E5‐2687W (3.1 GHz) and 252 GB RAM.

#### 2.5.1 Standardized T_1_/T_2_ phantom study

A standardized T_1_/T_2_ phantom (T1MES)^41^ was acquired together with 2 bottles of corn oil. Imaging parameters for Dixon‐cMRF included: spatial resolution 2 × 2 mm^2^, slice thickness = 8 mm, receiver bandwidth (BW) = 868 Hz/pixel, FOV = 512 × 512 mm^2^, TE1/TE2/TE3/TR = 2/3.6/5.2/7.5 ms, mid‐diastolic acquisition window of 188 ms, simulated heart rate of 60 bpm, 375 radial spokes, and *Nread* = 256 samples along the readout direction acquired in total for each TE, acquisition time ~15 s. Fully sampling k‐space in the Nyquist sense requires the acquisition of $\frac{\pi }{2}Nread$ radial spokes leading to an undersampling of R ~402 for each contrast acquired.

The single echo cMRF (echo 1) and water maps obtained from Dixon‐cMRF were compared to inversion‐recovery spin echo (IRSE) and T_2_ multi‐echo spin echo (MESE) reference measurements. Acquisition parameters included Cartesian readout, spatial resolution 2 × 2 mm^2^, slice thickness = 8 mm, 9 inversion times from 50 to 3000 ms, TR = 7000 ms for the IRSE experiment, and TR/TE/ΔTE = 7000/15/15 ms with 8 echoes for the MESE experiment.

#### 2.5.2 Water–fat phantom study

A water–fat phantom was built in‐house with 6 vials composed of a mix of different concentrations of a water solution and peanut oil, 1 vial of distilled water and agar (2%), and 1 vial of exclusively peanut oil. The peanut oil was chosen as it has a similar spectrum to the one found in the triglyceride protons present in human fat tissues.^42^ The water solution contained 43 mM sodium dodecyl sulfate (SDS), 43 mM sodium chloride, and 0.3 mM gadobenate dimeglumine (MultiHance, Bracco, Milan, Italy) as described in Hines et al^43^ and agar (2%) for solidification.

A proton density fat fraction (PDFF) reference measurement was made using a 6‐echo Dixon GRE sequence. Acquisition parameters included: Cartesian read‐outs with fly‐back, 6 echoes with TR/TE1/ΔTE = 13.7/1.3/2 ms, flip angle (FA) = 5°, BW = 1085 Hz/pixel, resolution = 2 × 2 mm, and slice thickness = 8 mm. The reference PDFF estimation uses the same pre‐defined 6‐peak fat model as used in the proposed Dixon‐cMRF.^37^ A graph cut scheme was considered for B_0_ estimation,^44^ and $T_{2}^{*}$ decay and noise bias correction^40^ were included for accurate PDFF estimation.

Dixon‐cMRF measurements were performed with the same acquisition parameters as in the standardized T_1_/T_2_ phantom study. Reference T_1_ IRSE and T_2_ MESE water scans were acquired with the same acquisition parameters as in the standardized T_1_/T_2_ phantom study but with binomial 1331 water excitation pulses to compare with Dixon‐cMRF water T_1_ and T_2_ maps. SASHA, MOLLI, and T_2_‐GRASE sequences were also acquired to compare the performance of conventional techniques in the presence of water–fat partial volume. Acquisition parameters for SASHA included: TE/TR = 1.19/2.4 ms, SENSE factor = 2, BW = 1085 Hz/pixel, FA = 70°, 9 saturation times ~120:60:650 ms and an infinity image, and acquisition time = 10 s. Acquisition parameters for MOLLI (5(3)3) included: TE/TR = 1.19/2.4 ms, SENSE factor = 2, BW = 1085 Hz/pixel, FA = 35°, and acquisition time = 11 s. T_2_‐GRASE acquisition parameters included: 9 TEs = 8.3:8.3:74.7 ms, EPI factor = 7, FA = 90°, SENSE factor = 2.4, double inversion recovery for blood signal nulling and acquisition time = 21 s.

#### 2.5.3 In vivo study

In vivo acquisitions were performed under a single breath‐hold of ~15 s with the same parameters used for the phantom experiments in short‐axis orientation. Conventional SASHA, MOLLI, and T_2_‐GRASE maps were acquired sequentially with Cartesian readouts and the same FOV, resolution, and slice thickness as Dixon‐cMRF in all subjects. Additionally, PDFF reference measurement was made using a 6‐echo Dixon GRE sequence in 3 of 10 subjects. SASHA, MOLLI, and T_2_‐GRASE acquisition parameters were as described in the water–fat phantom study. Six‐point Dixon acquisition and reconstruction parameters were the same as those used for the water–fat phantom experiment but with SENSE factor = 2 and acquisition time ~10 s.

Dixon‐cMRF time series images were reconstructed using HD‐PROST. Additionally, LRI, LRI with locally low‐rank and Wavelet regularization (SLLR),^45^ and HD‐PROST reconstructions were performed in a representative healthy subject for comparison purposes.

### 2.6 Analysis

T_1_ and T_2_ values were measured in region of interests (ROIs) for the phantoms and in vivo experiments. Mean values within the ROI were used to assess accuracy whereas the SD within the ROI (spatial variability) was considered as an indication of the mapping precision in phantom^46^ and used as a surrogate for precision in the healthy myocardium measurements.^10^ Accuracy of T_1_ and T_2_ water Dixon‐cMRF was assessed in the standardized T_1_/T_2_ phantom study through lines of best fit, determination coefficient, and normalized RMSE (NRMSE) with respect to reference IRSE and MESE measurements. Dixon‐cMRF FF estimation was compared to the reference PDFF measurement in the water–fat phantom study and in 3 healthy subjects through determination coefficients and maximum error. Rigid registration between the PDFF and Dixon‐cMRF FF maps was performed previous to the analysis of the in vivo data. In vivo T_1_ and T_2_ measurements were performed in the septum, and using the 6 AHA cardiac segments model, compared against MOLLI, SASHA, and T_2_‐GRASE. A 2‐tailed Student’s t test with Bonferroni correction (resulting threshold *P*‐values were *P*_T1_ < 0.025 and *P*_T2_ < 0.05 for T_1_ and T_2_ measurement methods, respectively) was performed on the mean and spatial variability septum measurements to test for statistically significant differences between the methods. Dixon‐cMRF FF was measured in the septum for all subjects and additionally in 2 separate pericardial (pericardial 1 and pericardial 2) and 1 subcutaneous fat ROIs for the 3 subjects that underwent PDFF experiments.

In vivo Dixon‐cMRF water T_1_ and T_2_ maps and conventional MOLLI, SASHA, and T_2_‐GRASE maps were qualitatively assessed by an expert (R.H.) with 1 year of expertise in cardiac T_1_ and T_2_ mapping using a 4‐point scoring system: 1, uninterpretable maps; 2, poor map quality (blurred edges, noise, and residual artefacts); 3, acceptable map quality (mildly blurred edges, mild noise, and residual artefacts); and 4, excellent map quality (sharply defined myocardium wall, neglectable residual artefacts). A Wilcoxon signed rank test with Bonferroni correction (*P*_T1_ < 0.025 and *P*_T2_ < 0.05) was performed to test for statistically significant differences between the scores.

Water and fat T_1_ and T_2_ Dixon‐cMRF values within a partial volume mask were compared to those obtained with single echo cMRF for each of the 3 echoes to investigate any potential water–fat partial volume bias. The locations of the pixels surrounding the heart affected by water–fat partial volume, to define the mask, were extracted using the Dixon‐cMRF fat fraction map (FF ϵ [0.3; 0.7]).

## 3 RESULTS

### 3.1 Standardized T_1_/T_2_ phantom study

**Figure 2:**
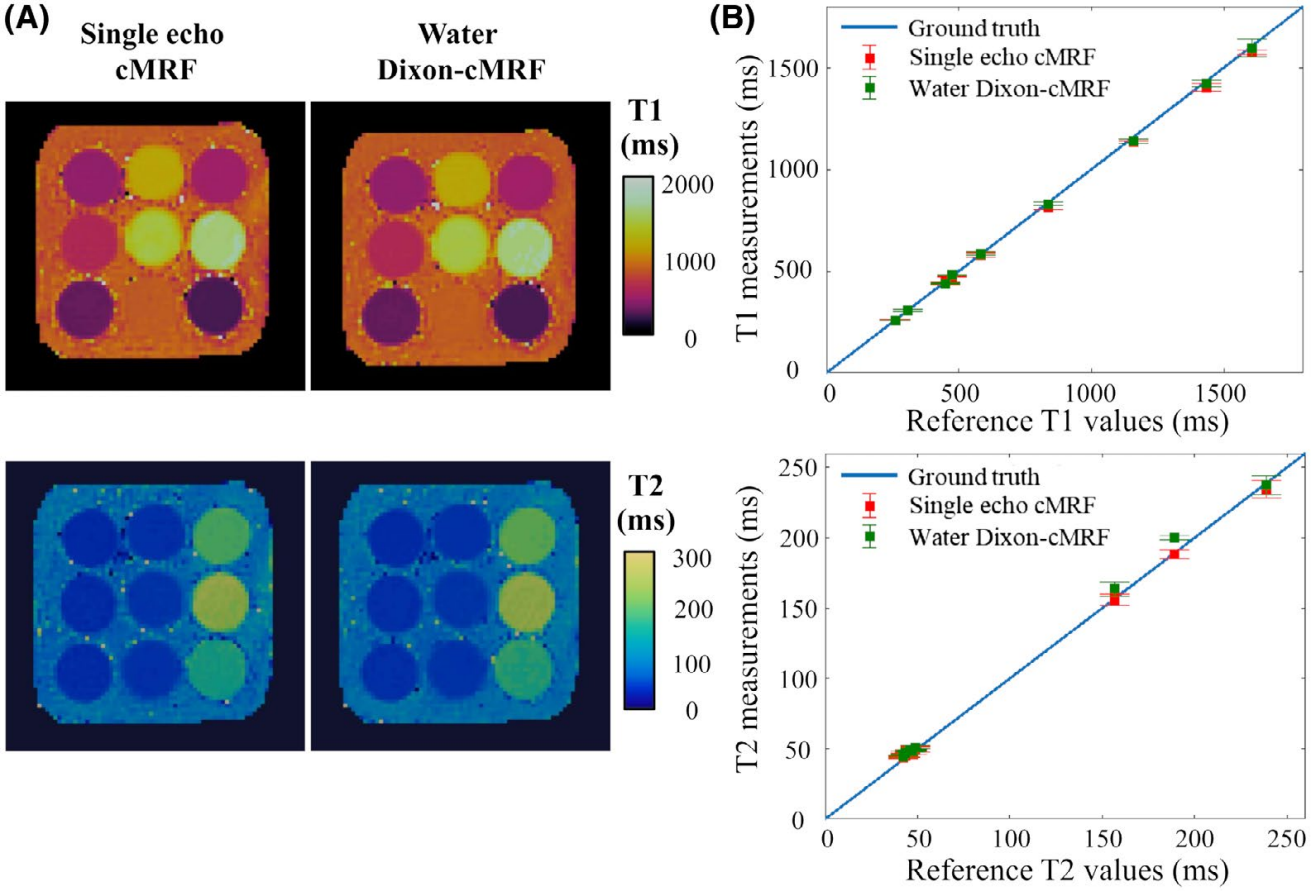
Dixon‐cMRF T_1_/T_2_ phantom experiment. (A) Single echo cMRF (echo 1) and Dixon‐cMRF water‐specific T_1_ and T_2_ maps. (B) T_1_ and T_2_ values for all vials measured with water Dixon‐cMRF and single echo cMRF (echo 1) in comparison to reference spin echo values. For both single echo cMRF and water Dixon‐cMRF high determination coefficient (R^2^ ≥ 0.99) and low NRMSE (~5%) were obtained when compared with reference values

Dixon‐cMRF results obtained on the standardized T_1_/T_2_ phantom are summarized in Figure 2. Dixon‐cMRF water T_1_ and T_2_ values are in good agreement with the spin‐echo reference values and those estimated with single echo cMRF (echo 1). Lines of best fit with slopes a_T1_ = 0.975/0.990 and a_T2_ = 0.972/1.008, intercepts of b_T1_ = 5.8/3.6 ms and b_T2_ = 2.5/2.5 ms, and high determination coefficients (both R^2^ > 0.99) with respect to the reference values were obtained for single echo cMRF (echo 1) and water Dixon‐cMRF, respectively. NRMSE over all vials were measured with single echo cMRF and water Dixon‐cMRF maps at 1.9% and 1.4% for T_1_ measurements and 3.6% and 5.4% for T_2_ measurements, respectively. In Supporting Information Figure S3, a good water–fat separation is observed visually in both water and fat M_0_ maps as well as a smooth off‐resonance map.

### 3.2 Water–fat phantom study

**Figure 3:**
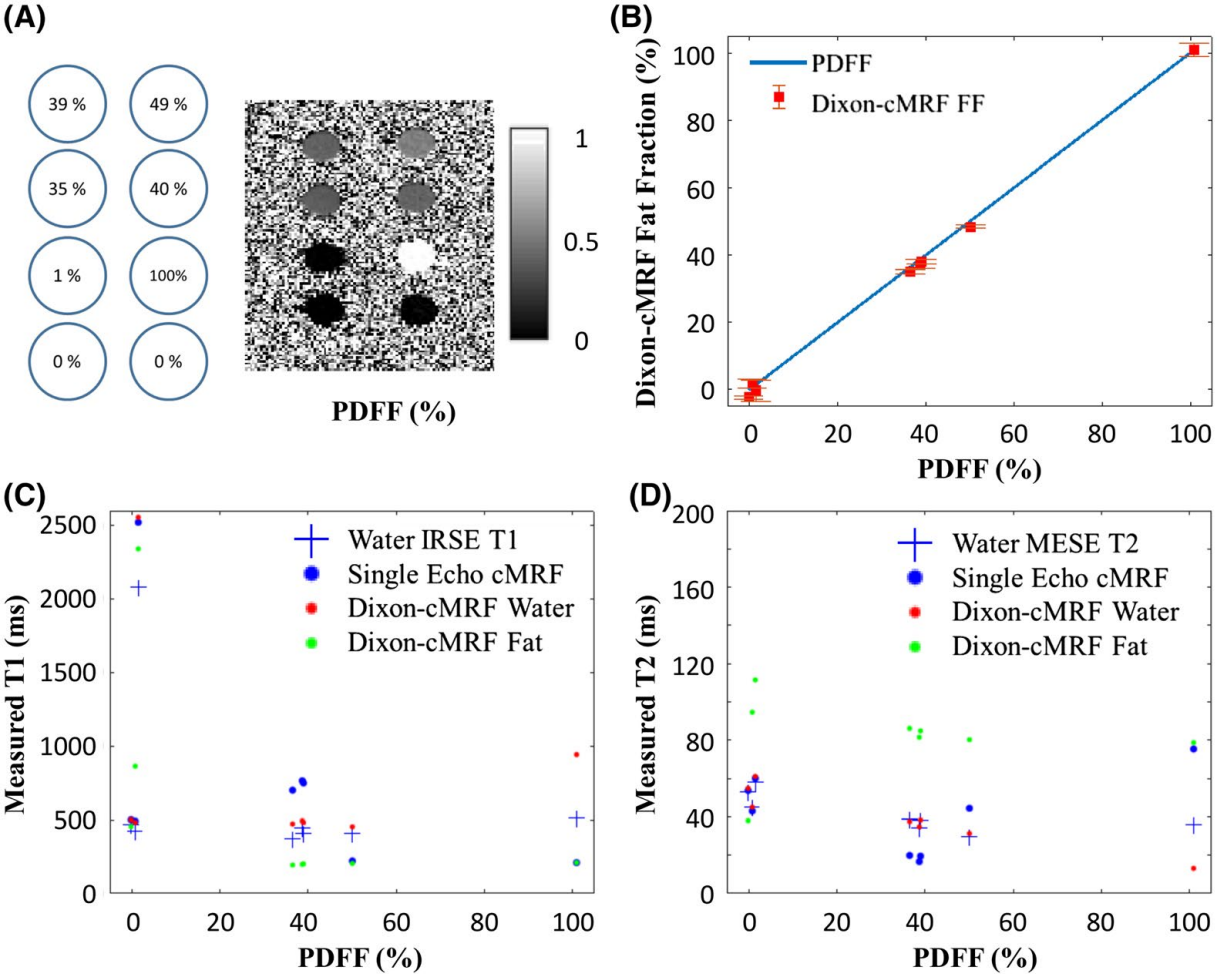
(A) Reference in‐house phantom values and map measured with proton density fat fraction (PDFF). (B) Dixon‐cMRF fat fraction (FF) measurement compared to the reference PDFF for all phantom vials. (C) T_1_ estimation using single echo cMRF (echo 1) and Dixon‐cMRF (water‐ and fat‐specific) in comparison to the reference water selective IRSE acquisition. (D) T_2_ estimation using single echo cMRF (echo 1) and Dixon‐cMRF (water‐ and fat‐specific) in comparison to the reference water‐selective T_2_ MESE measurement

Reference PDFF measurements for the water–fat phantom are shown in comparison to Dixon‐cMRF FF estimation in Figure 3A and B. A high determination coefficient of R^2^ = 0.999 was obtained between both measurements, with a maximum NRMSE of 2.1% (observed at very low FF). Dixon‐cMRF water and fat T_1_ and T_2_ measurements are shown in Figure 3C and D in comparison to water‐excited spin echo and single echo cMRF (echo 1) measurements. The corresponding maps are shown in Supporting Information Figure S4. High determination coefficients were observed between water Dixon‐cMRF and the reference values (excluding the fat only vial) with R^2^ = 0.999 and R^2^ = 0.992 for T_1_ and T_2_, respectively. A maximum error with respect to references in the presence of water–fat partial volume was measured at 100 ms and 3 ms for T_1_ and T_2_, respectively. Water and fat Dixon‐cMRF T_1_ and T_2_ values are compared to MOLLI, SASHA, and T_2_‐GRASE measurements in Supporting Information Figure S5A and to single echo cMRF for each of the 3 echoes in Supporting Information Figure S5B. A dependency of the original cMRF measurement on the echo time selected in the presence of water fat partial volume was observed. Echo 1, which is closest to out‐of‐phase (2 ms), provided particularly poor matches in the presence of water–fat partial volume.

### 3.3 In vivo study

Dixon‐cMRF water T_1_ and T_2_ maps and FF estimation obtained with LRI, SLLR, and HD‐PROST reconstructions are shown in Supporting Information Figure S6. Results show remaining noise‐like artefacts in the unregularized LRI maps, whereas regularized SLLR and to a greater extent HD‐PROST provided excellent quality water T_1_ and water T_2_ maps (scored 4 for HD‐PROST) as well as visually good FF maps.

**Figure 4:**
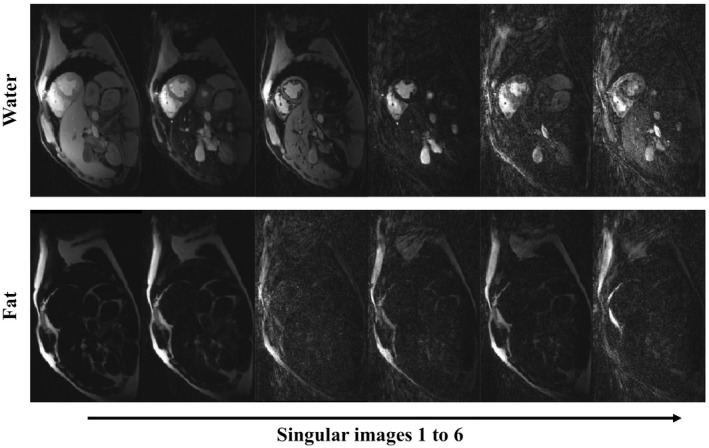
Water–fat separated Dixon‐cMRF singular images for a representative healthy subject

**Figure 5:**
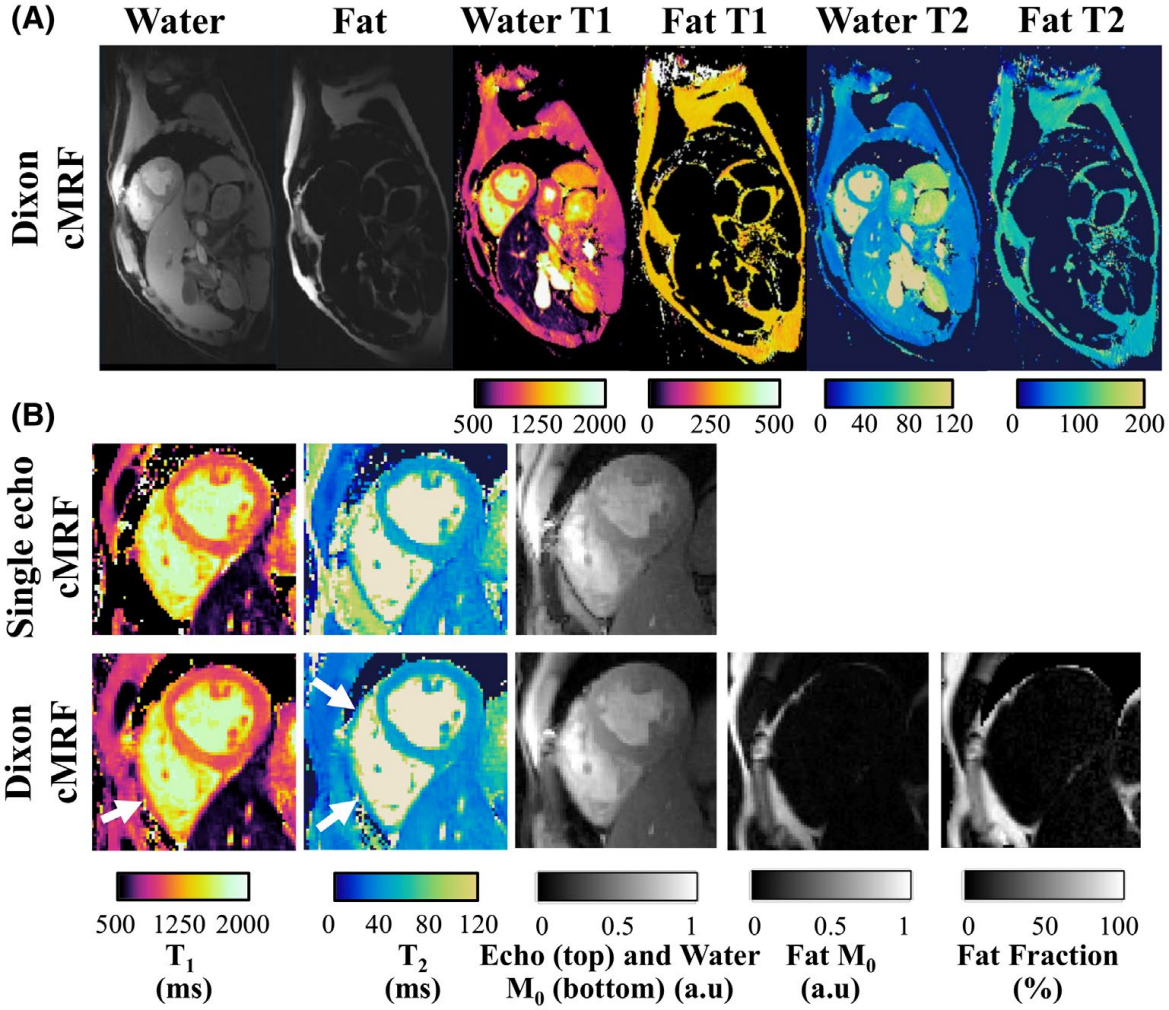
Dixon‐cMRF in a representative healthy subject. (A) Good image quality and water–fat separation is seen on the whole FOV of the 1st singular images leading to good quality water and fat T_1_ and T_2_ maps. (B) Comparison of Dixon‐cMRF and single echo cMRF (echo 1) in a zoomed region around the heart. Myocardium wall recovery with Dixon‐cMRF in both T_1_ and T_2_ maps in regions with water–fat partial volume is indicated by the white arrows. Additional water‐ and fat‐specific M_0_ can be estimated with Dixon‐cMRF to obtain a fat fraction map

Water–fat separation of the compressed Dixon‐cMRF time‐series (singular images) are shown for a representative healthy subject in Figure 4. The resulting water and fat T_1_ and T_2_ Dixon‐cMRF maps are shown in Figure 5. A zoom‐in region around the heart shows that Dixon‐cMRF recovers myocardium wall T_1_ and T_2_ information in the presence of fat partial volume in comparison to single echo cMRF (arrows). Dixon‐cMRF water T_1_ and T_2_ maps and FF estimation for 5 other subjects are shown in Supporting Information Figure S7.

**Figure 6:**
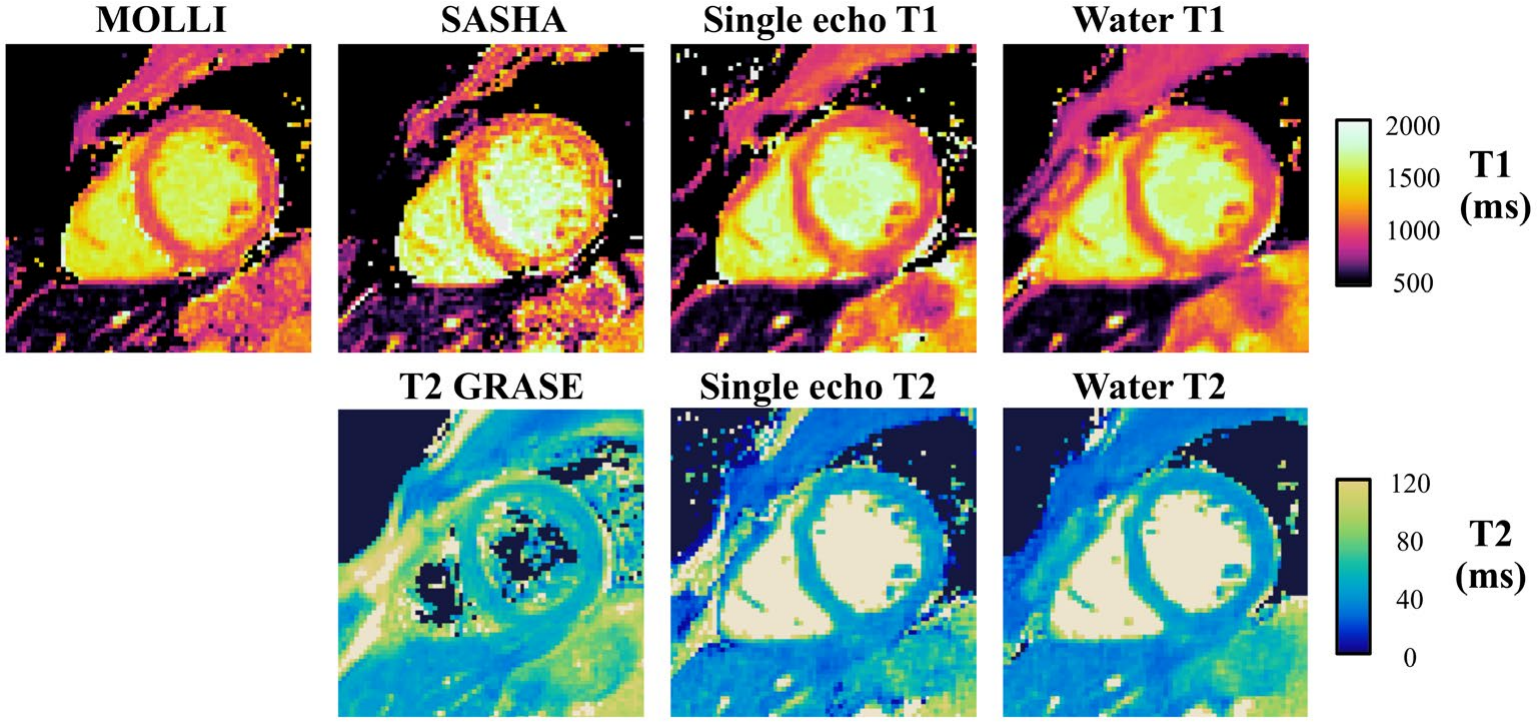
Comparison of water Dixon‐cMRF, single echo cMRF (echo 1), and conventional MOLLI, SASHA, and T_2_‐GRASE maps for a representative healthy subject

**Figure 7:**
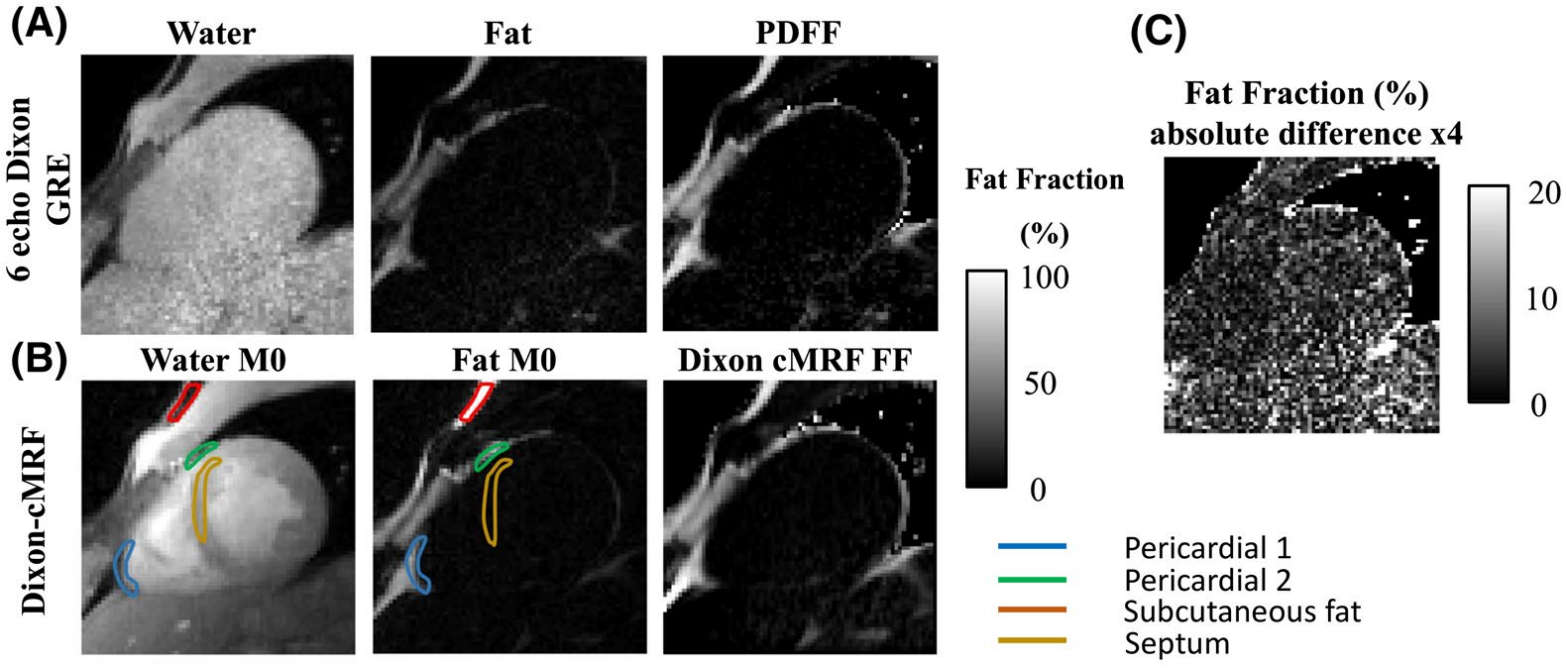
(A) Water and fat magnitude images and reference proton density fat fraction (PDFF) map obtained from a 6 echo Dixon GRE Cartesian scan. (B) Dixon‐cMRF water and fat M_0_ images as well as the resulting fat fraction (FF) estimate. The ROIs used for the analysis in Supporting Information Table S1 and Figure S9 are shown for this particular volunteer superimposed on the water M_0_ and fat M_0_ images. (C) Absolute difference image between the reference PDFF and Dixon‐cMRF FF maps

Dixon‐cMRF water T_1_ and T_2_ maps are shown in Figure 6 in comparison to MOLLI, SASHA, T_2_‐GRASE, and single echo cMRF (echo 1). Comparable results are observed with all methods, however, the proposed Dixon‐cMRF provides additional FF estimation. Dixon‐cMRF water and fat M_0_ images and FF map are shown in Figure 7 together with the 6‐point Dixon PDFF reference. The absolute difference image between the PDFF and Dixon‐cMRF FF maps is also included (Figure 7C).

**Figure 8:**
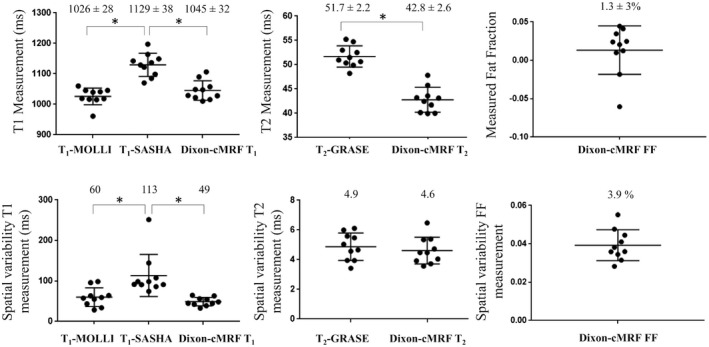
Top: T_1_, T_2_, and FF septum measurements in 10 healthy subjects for the different mapping techniques. Average across subjects ± SD (ms) are reported for each technique on the top of each plot. Bottom: spatial variability on the T_1_, T_2_, and FF septum measurements for the different mapping techniques. Average spatial variability (ms) of the measurements are reported for each technique on the top of each plot. Differences with statistical significance are identified by * (*P* < 0.025 for T_1_ and *P* < 0.05 for T_2_)

Qualitative map quality median scores of 2, 3, and 3.5 were obtained for SASHA, MOLLI, and Dixon‐cMRF water T_1_, respectively, and of 3 and 4 for T_2_‐GRASE and Dixon‐cMRF water T_2_, respectively. The difference between scores were not significant between Dixon‐cMRF T_1_ and MOLLI (*P* = 0.125), whereas MOLLI (*P* = 0.002) and Dixon‐cMRF T_1_ (*P* = 0.002) were both scored significantly better than SASHA. Dixon‐cMRF T_2_ was also scored significantly better than T_2_‐GRASE (*P* = 0.0156). The distribution of the map quality scores for each mapping technique is shown in Supporting Information Figure S8. Dixon‐cMRF water T_1_, T_2_, and FF septum measurements (mean and spatial variability) are shown in Figure 8 for all subjects, in comparison to measurements from SASHA, MOLLI, and T_2_‐GRASE. The average T_1_ septum measurements using SASHA, MOLLI, and the proposed water Dixon‐cMRF T_1_ were 1129 ± 38 ms, 1026 ± 28 ms, and 1045 ± 32 ms, respectively. The average T_2_ measurements for T_2_‐GRASE and the proposed water Dixon‐cMRF T_2_ were 51.7 ± 2.2 ms and 42.8 ± 2.6 ms, respectively. The bias between the mean of Dixon‐cMRF T_1_ and T_2_ measurements was −84 ms and −8.9 ms with respect to SASHA and T_2_‐GRASE, respectively, differences were statistically significant in both cases (*P* < 0.0001). The average Dixon‐cMRF septum FF value was 1.3 ± 3% across all subjects.

**Figure 9:**
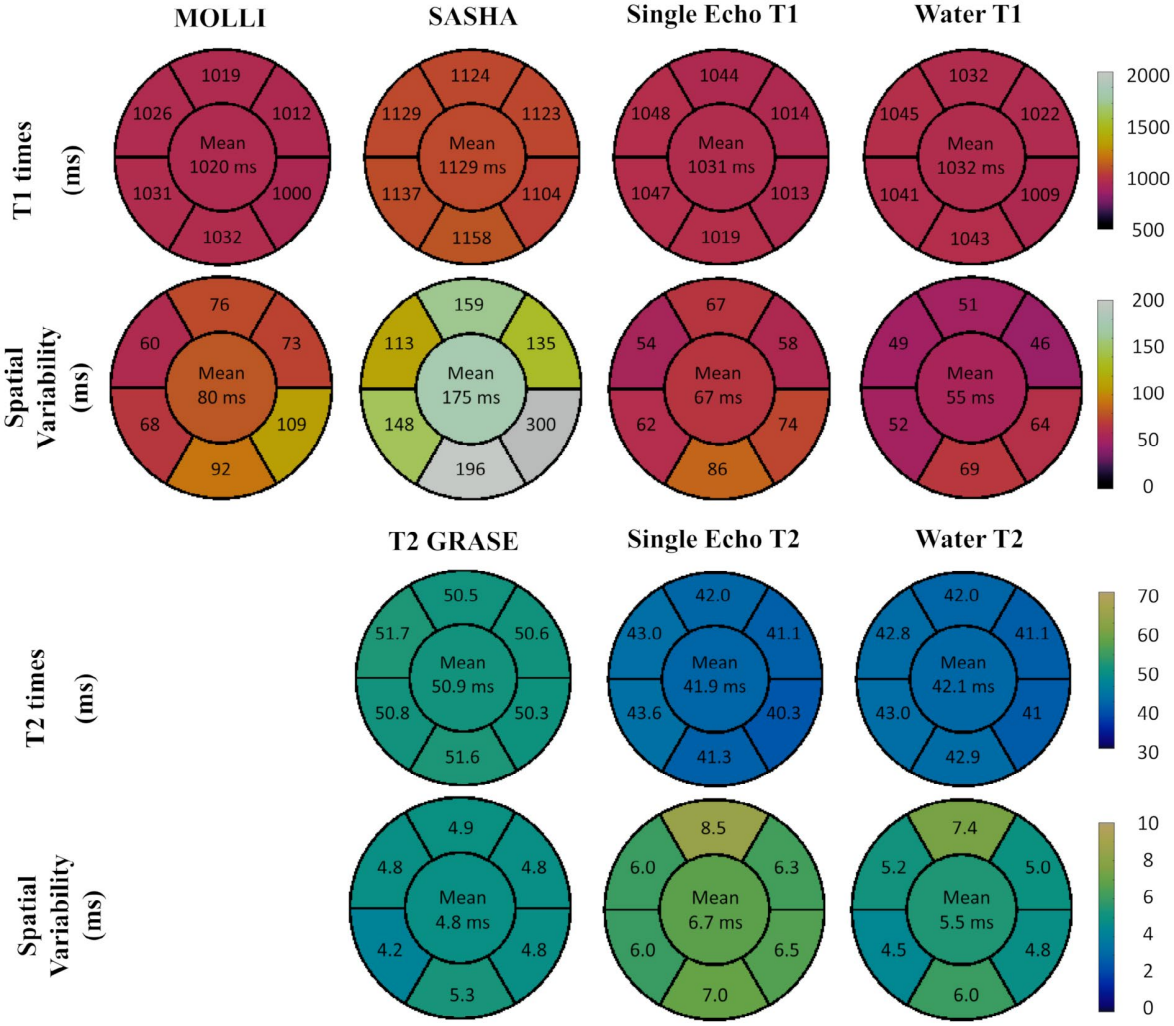
Regional T_1_ and T_2_ assessment of the different mapping techniques. T_1_ (top) and T_2_ (bottom) mean values and spatial variability reported for each segment as average measurements over 10 subjects. The value in the central segment represents the average over all segments. Average measurements/spatial variability for Dixon‐cMRF water T_1_ and T_2_ were 1032/55 ms and 42.1/5.5 ms, respectively. Overall Dixon‐cMRF presented lower spatial variability than MOLLI and SASHA measurements but higher than T_2_‐GRASE measurements

In the septum, Dixon‐cMRF water T_1_ mapping achieved lower spatial variability (49 ms) than MOLLI (60 ms) and SASHA (113 ms), whereas Dixon‐cMRF water T_2_ mapping spatial variability (4.6 ms) was similar to T_2_‐GRASE (4.9 ms). Dixon‐cMRF FF spatial variability in the septum was 3.9%. Regional 6‐segment T_1_ and T_2_ mean measurements and spatial variability across all subjects are shown in Figure 9 for the proposed Dixon‐cMRF water T_1_ and T_2_, in comparison to MOLLI, SASHA, T_2_‐GRASE and single echo cMRF (echo 1).

PDFF and the Dixon‐cMRF FF measurements for 4 ROIs (pericardial 1, pericardial 2, subcutaneous fat, and septum) are shown in Supporting Information Table S1 and Figure S9 for the 3 subjects that underwent PDFF acquisition. Good agreement was observed between the reference PDFF and the Dixon‐cMRF FF measurements (R^2^ > 0.98), with a maximum absolute difference of 5.8% FF observed for low fat fractions.

**Figure 10:**
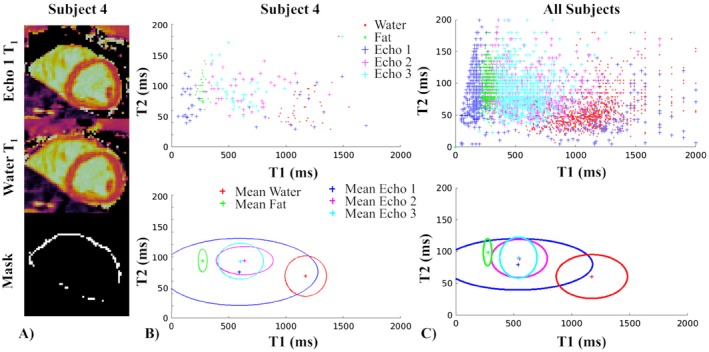
(A) Single echo (echo 1) cMRF T_1_, Dixon‐cMRF water T_1_ and water–fat partial volume mask (with FF ϵ [0.3; 0.7]). The mask was used to obtain the T_1_ and T_2_ values of single echo cMRF for echoes 1, 2, 3, and Dixon‐cMRF water and fat maps that are shown in (B) as point clouds (top) and ellipses (bottom). The ellipse is centered on the mean and the horizontal and vertical radius represent the SD over T_1_ and T_2_ measurements, respectively. (C) Same plots in (B) using the values obtained from all the subjects included in the study. The 2 compartments of water and fat are clearly separated in (B) and (C). Mean T_1_ and T_2_ measurements in the masked pixels for water are 1174 ms and 60.9 ms, respectively, whereas mean values for single echo cMRF for each of the 3 echoes are: echo 1 T_1_/T_2_ = 537/80.1 ms, echo 2 T_1_/T_2_ = 549/89.3 ms, and echo 3 T_1_/T_2_ = 541/90.6 ms

The water–fat partial volume mask used to compare water and fat Dixon‐cMRF and single echo cMRF (for each echo) T_1_ and T_2_ values in the presence of partial volume is shown Figure 10A for a representative healthy subject. The distribution of the corresponding measurements within the mask are shown in Figure 10B for the same healthy subject and in Figure 10C for all subjects. The 2 compartments of water and fat are clearly separated for the proposed Dixon‐cMRF. Mean water T_1_ and T_2_ values, for the pixels within the partial volume mask, were 1174 ms and 60.9 ms, respectively, whereas mean values for single echo cMRF for each of the 3 echoes were: echo 1 T_1_/T_2_ = 537/80.1 ms, echo 2 T_1_/T_2_ = 549/89.3 ms, and echo 3 T_1_/T_2_ = 541/90.6 ms.

## 4 DISCUSSION

A novel 3‐point Dixon cardiac MRF framework was proposed to enable simultaneous water‐ and fat‐specific T_1_, T_2_, and M_0_ mapping of the heart and fat fraction estimation in a single ~15s breath‐hold scan per slice. This is achieved by extending cardiac MRF using a 3‐echo radial acquisition, a water–fat separation of the MRF time series images in the compressed temporal domain and the recently introduced multi‐contrast HD‐PROST reconstruction.

### 4.1 T_1_ and T_2_ quantification

T_1_ and T_2_ quantification with the proposed Dixon‐cMRF framework was validated in a standardized T_1_/T_2_ phantom. Accurate water T_1_ and T_2_ values were obtained with the proposed approach in comparison to spin echo‐based reference values (R^2^ > 0.99, NRMSE_T1_ = 1.4% and NRMSE_T2_ = 5.4%). A slice profile correction was included in the dictionary to obtain accurate measurements (for T_2_ especially) when using short pulses.^27^, ^39^ A Bloch simulation of the inversion efficiency was also included,^27^ impacting mainly the T_1_ measurements. To limit the impact of B_1_ inhomogeneities, adiabatic inversions^47^ and refocusing pulses in the T_2_ preparation pre‐pulses^48^ were used, with the maximum excitation flip angle^27^ limited to 30°. To extend the pattern from single echo to 3‐echo Dixon encoding, the TEs were chosen based on a previously reported Cramer‐Rao lower bound (CRLB) optimization.^49^ However, improvements may be possible, because this CRLB optimization was performed considering a single peak model, no $T_{2}^{*}$ decay, and no signal variations due to MRF.

Dixon‐cMRF water T_1_ and T_2_ maps were judged as being of good or excellent quality (score of 3 or more) in all the healthy subjects in this study. In vivo Dixon‐cMRF water T_1_ values were slightly higher than those measured with MOLLI but lower than SASHA, which is consistent with previously reported results for cMRF,^27^ whereas Dixon‐cMRF water T_2_ values were lower than T_2_‐GRASE measurements. Discrepancies between previously reported T_2_ values for different T_2_ mapping methods (T_2_‐GRASE vs. T_2_ prep bSSFP) and field strengths (1.5T vs. 3T)^50^ hinders further analysis of this bias. However, myocardium measurements previously reported for cardiac MRF^13^, ^27^ seem to be in the lower end of existing literature values. Magnetization transfer and diffusion have previously been shown to cause considerable biases in MRF^51^, ^52^, ^53^ and might be the source of the remaining biases seen in the in vivo measurements. T_1_, T_2_, and M_0_ measurements in the blood are also susceptible to flow as blood entering and leaving the imaging slice could not be accurately modeled. Validation of Dixon‐cMRF water T_1_ and T_2_ measurements in the presence of water–fat partial volume could not be performed in vivo because of unavailability of robust water T_1_ and T_2_ reference mapping techniques, therefore this validation was performed on a water–fat phantom, as discussed below.

### 4.2 Water–fat separation and fat fraction estimation

Effective water–fat separation was obtained with the proposed Dixon‐cMRF in the water–fat phantom (Figure 3) and in vivo (Figures 4 and 5) experiments. High determination coefficients (R^2^ > 0.99 in the phantom and R^2^ > 0.98 in vivo) were observed between the FF map estimated with the proposed approach and the reference PDFF map, with maximum errors obtained at very low FF values. In vivo septum average FF was measured at 1.3% that is in agreement with reference literature values (~1%).^54^ A limitation of the proposed Dixon‐cMRF approach is that $T_{2}^{*}$ decay is not accounted for in the water–fat separation model. The signal loss because of $T_{2}^{*}$ decay can be accounted for as a proportion of the signal coming from an off‐resonant fat compartment. This effect has been previously shown to impact FF estimation especially at low FF values and short $T_{2}^{*}$.^43^ Negative fat fraction values were estimated in very low fat fraction areas in 2 subjects and in the phantom. The lack of $T_{2}^{*}$ correction^55^ and phase errors^56^ could be the main sources of these errors and will be investigated in future works. Extending the proposed approach to obtain additional $T_{2}^{*}$ mapping could provide more accurate FF values and $T_{2}^{*}$ quantification, which is relevant in the diagnosis and treatment monitoring of myocardial iron overload.^57^ Recently, a 3‐echo GRE MRF acquisition with considerably longer acquisition times was proposed for brain imaging, obtaining additional $T_{2}^{*}$ and $T_{2}^{\prime }$ maps.^58^ Clinical recommendations are to acquire 8 echoes from 2–18 ms for cardiac $T_{2}^{*}$ mapping,^59^ therefore longer and more numerous TEs would be necessary with Dixon‐cMRF to successfully map these parameters, and therefore further investigation to enable T_1_, T_2_, $T_{2}^{*}$, and FF maps in a single breath‐hold scan is needed.

### 4.3 Water T_1_–T_2_ bias correction from water–fat partial volume

Dixon‐cMRF was able to recover the T_1_ and T_2_ of the water and fat compartments in the water–fat partial volume phantom, achieving more accurate results than those obtained with conventional mapping techniques (Supporting Information Figure S5A). Water Dixon‐cMRF T_2_ values were in good agreement with the water‐selective spin echo reference (maximum error = 3 ms), whereas a consistent overestimation was observed for Dixon‐cMRF T_1_ measurements in comparison to water T_1_ IRSE. The Dixon‐cMRF water T_1_ values with or without water–fat partial volume were all consistent, whereas water T_1_ IRSE measurements presented larger variations depending on the vials. In this study, water selective spin echo measurements were considered as reference measurements (because of the unavailability of a better gold standard), however, these measurements could be affected by remaining fat signals if the water selective pulse fails to fully suppress the different fat compartments. Additionally, the discrepancy between the reference water IRSE and Dixon‐cMRF T_1_ measurement (Figure 3C) for the distilled water vial (with the highest T_1_ value, outside of the range of interest for cardiac imaging) indicates that either the water IRSE (with maximum TI of 3000 ms) and/or the Dixon‐cMRF water measurement may not be able to map accurately very long T_1_ values, therefore further validations for very long T_1_ are needed.

Single echo cMRF behaved differently depending on the echo time in the presence of water–fat partial volume in the phantom experiment (Supporting Information Figure S5B). Similar observations were made in vivo by studying pixels within a water–fat partial volume mask, showing the dependency of the cMRF measurements on the echo time in zones affected by water–fat partial volume. The first echo (closest to out‐of‐phase: TE = 2 ms) was shown to provide inaccurate T_1_ and T_2_ values in zones with water–fat partial volume in both phantom and in vivo experiments. The water–fat separation used in the proposed Dixon‐cMRF allows to recover the T_1_ and T_2_ for the 2 (water and fat) compartments with observable wall recovery in water maps.

Good quantitative correspondences were observed in vivo between single echo cMRF (echo 1) and water Dixon‐cMRF T_1_ and T_2_ measurements in the septum (+7 ms and −0.2 ms bias for T_1_ and T_2_ measurements, respectively). A slightly lower spatial variability was observed in the water Dixon‐cMRF maps compared to single echo cMRF (echo 1) with an average spatial variability for Dixon‐cMRF and single echo cMRF of 55 ms and 67 ms, respectively, for T_1_, and of 5.5 ms and 6.7 ms for T_2_. This decrease in spatial variability was likely because of the SNR increase when combining the 3 echoes to obtain the water images.

### 4.4 Dixon‐cMRF in context of current MRF techniques

Dixon‐cMRF uses a water–fat separation of the MRF time series before dictionary matching of the water and fat compartments. Recently proposed dictionary‐based water–fat separation^22^, ^23^ could also be potentially applied to cardiac MRF, however, these methods significantly increase the dictionary size, require longer acquisition times than Dixon‐cMRF, and do not map T_2_ relaxation times, which makes it impractical for cardiac applications. Other works on chemical shift‐based approaches for MRF fat fraction estimation^24^, ^25^, ^60^ rely on long spiral readouts where water and fat deblurring will be necessary because of the long readouts. The advantage of the proposed Dixon‐cMRF over these techniques for cardiac imaging lies in its relatively short scan time, low sensitivity to nuisance parameters such as B_1_ and B_0_, and the use of short radial readouts (1.15 ms) leading to the fast acquisition of 3 echoes in the same TR with minimal blurring. Therefore, no extra field map acquisitions are necessary and the dictionary size remains unchanged compared to single echo cMRF. This is relevant in the context of cMRF where field maps would be acquired in separate breath‐holds leading to longer scan sessions and possible mis‐registration. A simplified analysis for solving the same problem using a dictionary‐based water–fat separation^23^ leads to 4 additional parameters in the dictionary (fat fraction, B_0_, T_1_ fat, and T_2_ fat). Considering the same discretization as in Cencini et al^23^ (dictionary 1), the 6D dictionary would have N_ff_ = 10, N_B0_ = 168, N_T1fat_ = 7, and for Dixon‐cMRF an added N_T2fat_ = 7 number of entries for FF, B_0_, T_1_ fat, and the additional T_2_ fat dimension, respectively. The dictionary size would therefore be multiplied by ~82,000 leading to ~10^9^ entries, 8500 days for dictionary computation (assuming linear increase of the computational complexity with the number of entries), and 36 h for matching (using an exhaustive search) with our current MATLAB implementation. In comparison, Dixon‐cMRF takes 2.5 h for dictionary computation, 0.3 s for water–fat separation, and 3.2 s for matching of water and fat.

Compared to previous works on both cMRF^13^, ^27^ and water–fat MRF,^23^, ^24^ Dixon‐cMRF reached higher acceleration rates to accommodate to the timing restrictions of cardiac MRI (ECG triggering, <200 ms acquisition window, and ~15 heartbeat breath‐holds) and multi‐echo Dixon imaging (acquisition of multiple echoes leading to longer TRs). The acquisition window of previously proposed cardiac MRF^13^ was 240–280 ms (16 heartbeats acquisition), which may lead to residual bias and artefacts because of cardiac motion. In this study, the acquisition window of Dixon‐cMRF was considerably shorter (188 ms leading to an effective scanning time of 2.82 s with 15 heartbeats acquisition) and similar to the clinical MOLLI sequence (180 ms).^4^

### 4.5 Study limitations and future work

Potential clinical applications for Dixon‐cMRF include, beside myocardial tissue characterization, patient populations where the detection of fat infiltration,^16^ volume quantification of pericardial^61^ and epicardial^19^ fat, and fat fraction values are of interest. An exemplary application is for patients with prior myocardial infarction, where detection of fat infiltration^16^ has high prognostic value and slight changes in acquisition parameters, such as the TE, might lead to potentially wrong diagnosis (depending on the bias induced by phase difference between fat and water).^18^ Dixon‐cMRF may allow to specifically identify whether the parameter variation is because of fat infiltration or another pathophysiology. In this study, only a small number of healthy subjects with no fat infiltration were acquired, spatial variability was used as a surrogate for precision, and no reference for the cardiac in vivo water specific T_1_ and T_2_ measurements was available. Further validation of the proposed Dixon‐cMRF technique in the presence of fat infiltrations (i.e., in patients with cardiovascular disease) will be investigated in future studies.

Finally, in this study, Dixon‐cMRF was performed without contrast agent injection. Acquiring Dixon‐cMRF both pre‐ and post‐contrast may allow to obtain additional extra cellular volume maps. The extension of Dixon‐cMRF to successfully map those additional parameters for global myocardial health assessment^14^ will be investigated in future work.

## 5 CONCLUSION

Dixon‐cMRF allows for simultaneous co‐registered quantification of myocardial water T_1_, water T_2_, and fat fraction in a single breath‐hold scan, enabling multi‐parametric T_1_, T_2_, and fat characterization. Moreover, reduced T_1_ and T_2_ quantification bias caused by water–fat partial volume was demonstrated in phantom experiments. Dixon‐cMRF has been successfully demonstrated in phantoms and healthy subject experiments. Clinical validation of this approach in patients with cardiovascular disease is now warranted.

## CONFLICT OF INTEREST

Dr. Doneva, Dr. Schneider, and Mr. Koken are Philips Healthcare employees.

## Supporting information


**FIGURE S1** HD‐PROST high‐order low rank regularization prior. The problem described in Equation 2 is solved using ADMM and is split into 2 sub‐problems: (1) data consistency with L2 regularization based on the denoised images obtained from solving the second sub‐problem and (2) high‐order SVD (HOSVD) denoising to enforce low‐rank regularization. For the second sub‐problem (shown in this figure), a tensor $T_{b}$ is assembled for the patch centered on voxel b by concatenating the K‐1 most similar patches within a neighborhood along the non‐local similarity dimension and the R contrasts along the spectral dimension. HOSVD is performed and the high‐order singular values are truncated according to the value of λ to produce a denoised tensor. This step is repeated for all the pixels in the multi‐contrast images. The final denoised multi‐contrast images are then obtained via aggregation and used as a prior in the sub‐problem 1 in the next iteration. Dixon‐cMRF reconstruction used 15 conjugate gradient iterations for the first sub‐problem and 6 ADMM iterations. Other reconstruction parameters were empirically set as number of patches K = 20, regularization λ = 0.001, patch size *N* = 5 × 5, and window search (neighborhood) = 20
**FIGURE S2** (A) Normalized magnitude of the singular values obtained from a singular value decomposition of the MRF dictionary in one representative healthy subject. (B) Curves describing the convergence of the algorithm for the images (x), prior (τ), and Lagrangian multiplier (y) of the 3 reconstructed echoes in function of the number of ADMM iterations for the same subject. (C) The resulting water T_1_ and T_2_ maps at different ADMM iterations showing the stability of the proposed Dixon‐cMRF reconstruction. A dictionary rank threshold of 6 (<3% of the first singular image) and 6 ADMM iterations were used for HD‐PROST reconstruction in this study
**FIGURE S3** Dixon‐cMRF T_1_/T_2_ phantom experiment. The standardized T1mes phantom was acquired together with 2 bottles of oil. (A) Water M_0_, fat M_0_, and B_0_ maps showing successful separation of water and fat signals. (B) Dixon‐cMRF water‐specific and single echo cMRF (echo 1) T_1_ and T_2_ maps. Because the fat signal is well‐suppressed, the water maps match to noise in the fat bottles
**FIGURE S4** Water–fat partial volume phantom T_1_, T_2_, and FF measurements. Dixon‐cMRF maps show good qualitative correspondence with reference water selective IRSE, water selective MESE, and 6 echo PDFF scan, whereas single echo cMRF (echo 1) measurements seems to map inconsistently in vials affected by water–fat partial volume as also shown in Figure 3 and Supporting Information Figure S5B
**FIGURE S5** Water–fat partial volume phantom experiment. (A) Comparison of water‐ and fat‐specific Dixon‐cMRF T_1_ to conventional SASHA and MOLLI (left), and water‐ and fat‐specific Dixon‐cMRF T2 to T2‐GRASE (right). Conventional methods are unable to accurately estimate T_1_ or T_2_ for either of the 2 (water and fat) compartments. (B) Comparison of water‐ and fat‐specific Dixon‐cMRF and single echo cMRF for each of the 3 independent echo measurements. T_1_ and T_2_ measurements in the presence of partial volume varies depending on the echo time because of different contributions of fat and water. In particular, echo 1 that is closest to out‐of‐phase, provides particularly poor matches in the presence of water–fat partial volume
**FIGURE S6** Comparison of non‐regularized LRI reconstruction (left), regularized using locally low rank and Wavelet priors (SLLR)^45^ (middle), and reconstructed using HD‐PROST (using high order low rank regularization). LRI shows remaining noise‐like artefacts that can be removed using SLLR and HD‐PROST. Although both provide good quality results, HD‐PROST maps seem slightly sharper as shown by the black arrows
**FIGURE S7** In vivo Dixon‐cMRF water T_1_, water T_2_, and fat fraction maps for 5 additional healthy subjects. High quality (score superior or equal to 3) water T_1_ and T_2_ maps were consistently obtained for all subjects
**FIGURE S8** Map quality evaluation of T_1_ (left) and T_2_ (right) mapping techniques according to a 4‐point scale (1 = uninterpretable maps to 4 = excellent map quality). Reported median scores are 2, 3, and 3.5 for SASHA, MOLLI, and Dixon‐cMRF water T_1_, respectively, and 3 and 4 for T_2_‐GRASE and Dixon‐cMRF water T_2_, respectively. All Dixon‐cMRF water maps were of acceptable or excellent quality (score superior or equal to 3) and obtained equal or higher scores than their conventional counterpart SASHA, MOLLI, and T_2_‐GRASE maps in this study. Image scores of the proposed method were significantly better when compared to SASHA (*P < 0.025) and T_2_‐GRASE (*P < 0.05). The difference in scores with MOLLI was not statistically significant (*P* = 0.07)
**FIGURE S9** Comparison between Dixon‐cMRF fat fraction and proton density fat fraction measured in 4 ROIs (2 separate pericardial regions, subcutaneous fat, and septum) for 3 healthy subjects. High determination coefficient R_2_ = 0.9885 was measured
**TABLE S1** Mean proton density fat fraction (PDFF) and Dixon‐cMRF fat fraction (FF) measured in 2 separate pericardial (pericardial 1, pericardial 2), septum, and subcutaneous fat ROIs in 3 healthy subjects (corresponding to the values plotted in Supporting Information Figure S9). Spatial variability of the measurement in the septum and subcutaneous fat, where homogeneous regions are expected, are also reported as a surrogate for precision. Pericardial ROIs were chosen in heterogeneous regions with varying water–fat partial volume, and therefore spatial variability would not be indicative of precision and therefore not reported here. Good agreement (R_2_ = 0.9885) was observed between the 2 methods and maximum absolute difference was measured at 5.8%
