# Supplementary material for: Water–fat Dixon cardiac magnetic resonance fingerprinting
Source: Magn Reson Med. 2019 Nov 18;83(6):2107–23. doi: 10.1002/mrm.28070 (PMC7064906; doi:10.1002/mrm.28070)
Supplement: Supplementary file 1 — FIGURE S1 HD‐PROST high‐order low rank regularization prior. The problem described in Equation 2 is solved using ADMM and is split into 2 sub‐problems: (1) data consistency with L2 regularization based on the denoised images obtained from solving the second sub‐problem and (2) high‐order SVD (HOSVD) denoising to enforce low‐rank regularization. For the second sub‐problem (shown in this figure), a tensor Tb is assembled for the patch centered on voxel b by concatenating the K‐1 most similar patches within a neighborhood along the non‐local similarity dimension and the R contrasts along the spectral dimension. HOSVD is performed and the high‐order singular values are truncated according to the value of λ to produce a denoised tensor. This step is repeated for all the pixels in the multi‐contrast images. The final denoised multi‐contrast images are then obtained via aggregation and used as a prior in the sub‐problem 1 in the next iteration. Dixon‐cMRF reconstruction used 15 conjugate gradient iterations for the first sub‐problem and 6 ADMM iterations. Other reconstruction parameters were empirically set as number of patches K = 20, regularization λ = 0.001, patch size N = 5 × 5, and window search (neighborhood) = 20 FIGURE S2 (A) Normalized magnitude of the singular values obtained from a singular value decomposition of the MRF dictionary in one representative healthy subject. (B) Curves describing the convergence of the algorithm for the images (x), prior (τ), and Lagrangian multiplier (y) of the 3 reconstructed echoes in function of the number of ADMM iterations for the same subject. (C) The resulting water T1 and T2 maps at different ADMM iterations showing the stability of the proposed Dixon‐cMRF reconstruction. A dictionary rank threshold of 6 (<3% of the first singular image) and 6 ADMM iterations were used for HD‐PROST reconstruction in this study FIGURE S3 Dixon‐cMRF T1/T2 phantom experiment. The standardized T1mes phantom was acquired together with 2 bottles o [file MRM-83-2107-s001.docx]

**SUPPORTING INFORMATION**

**Supporting Information Figure S1**


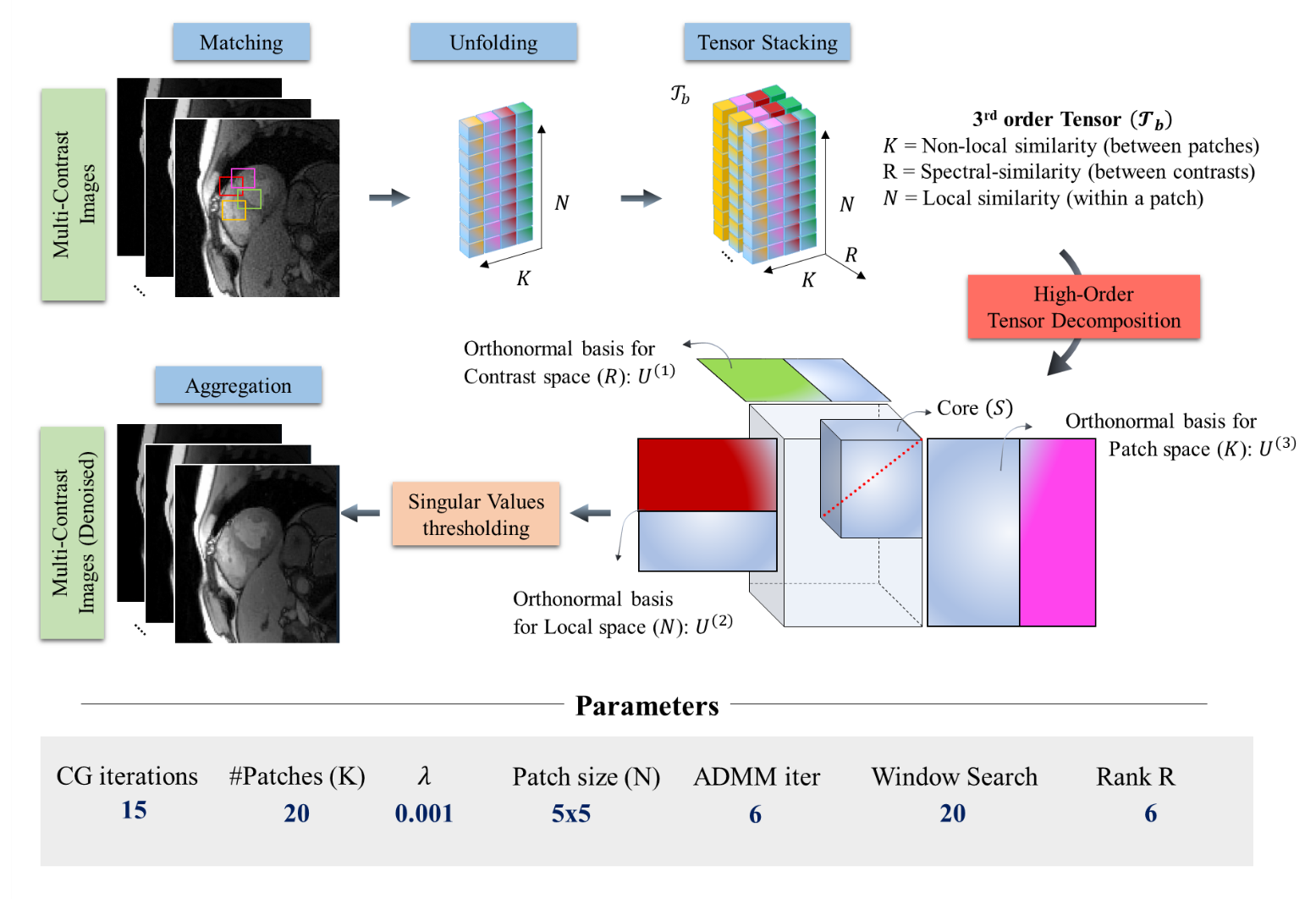


Supporting Information Figure S1. HD-PROST high-order low rank regularization prior. The problem described in Equation 2 is solved using ADMM and is split into 2 sub-problems: 1) data consistency with L_2_ regularization based on prior from the second sub-problem and 2) high-order SVD (HOSVD) denoising to enforce low-rank regularization. For the second sub-problem (shown in this figure), a tensor $\mathcal{T}_{b}$ is assembled for the patch centered on voxel b by concatenating the K-1 most similar patches within a neighborhood along the non-local similarity dimension, and the R contrasts along the spectral dimension. HOSVD is performed and the high-order singular values are truncated according to the value of $\lambda$ to produce a denoised tensor. This step is repeated for all the pixels in the multi-contrast images. The final denoised multi-contrast images are then obtained via aggregation and used as a prior in the sub-problem 1 in the next iteration. Dixon-cMRF reconstruction used 15 conjugate gradient iterations for the first sub-problem and 6 ADMM iterations. Other reconstruction parameters were empirically set as number of patches K = 20, regularization $\lambda=0.001$, patch size N=5x5 and window search (neighborhood) = 20.

**Supporting Information Figure S2**


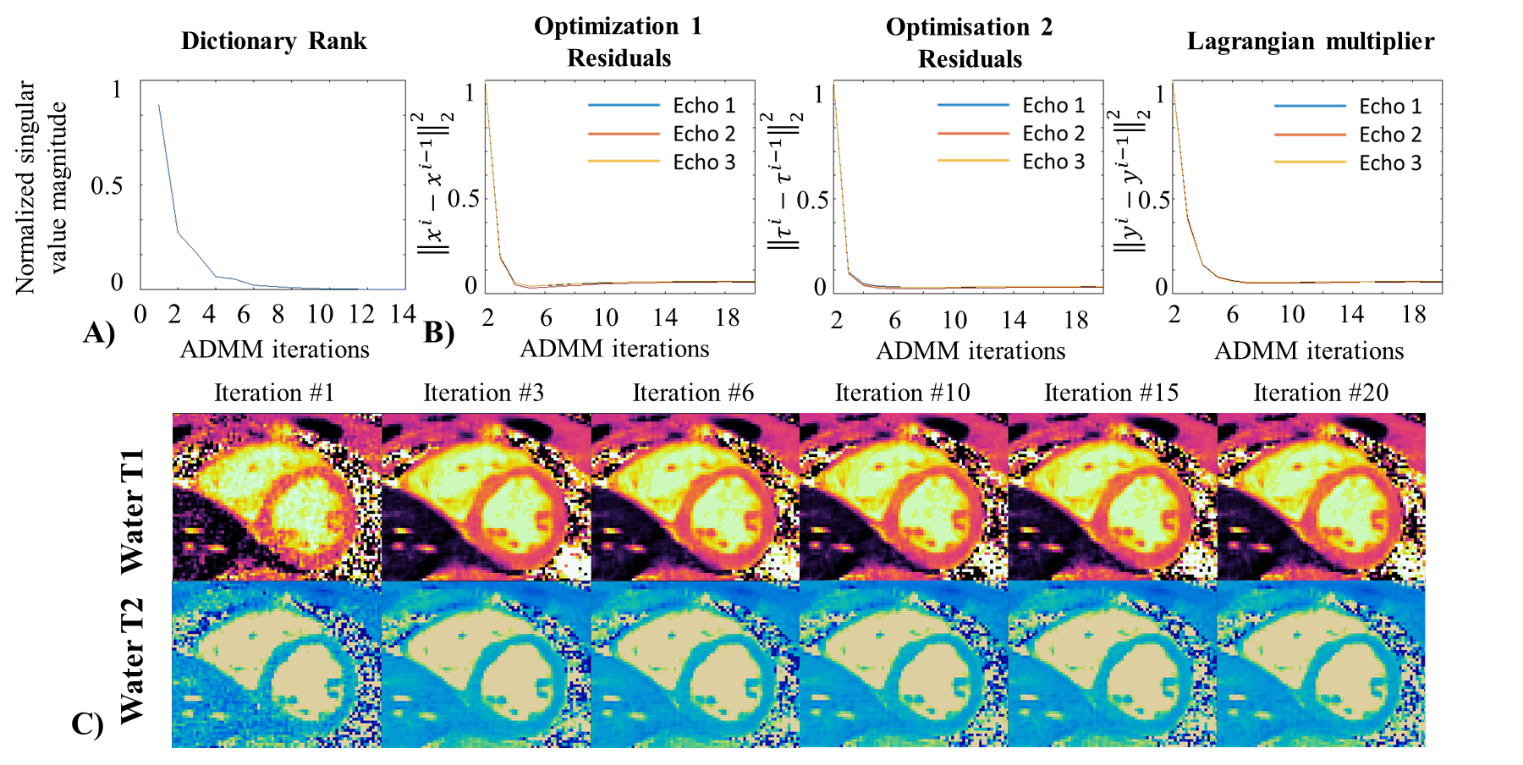


Supporting Information Figure S2. A) Normalized magnitude of the singular values obtained from a singular value decomposition of the MRF dictionary in one representative healthy subject. B) Curves describing the convergence of the algorithm for the images (x), prior (τ) and Lagrangian multiplier (y) of the three reconstructed echoes in function of the number of ADMM iterations, for the same subject. C) The resulting water T1 and T2 maps at different ADMM iterations showing the stability of the proposed Dixon-cMRF reconstruction. A dictionary rank threshold of 6 (<3% of the first singular image) and 6 ADMM iterations were used for HD-PROST reconstruction in this study.

**Supporting Information Figure S3**


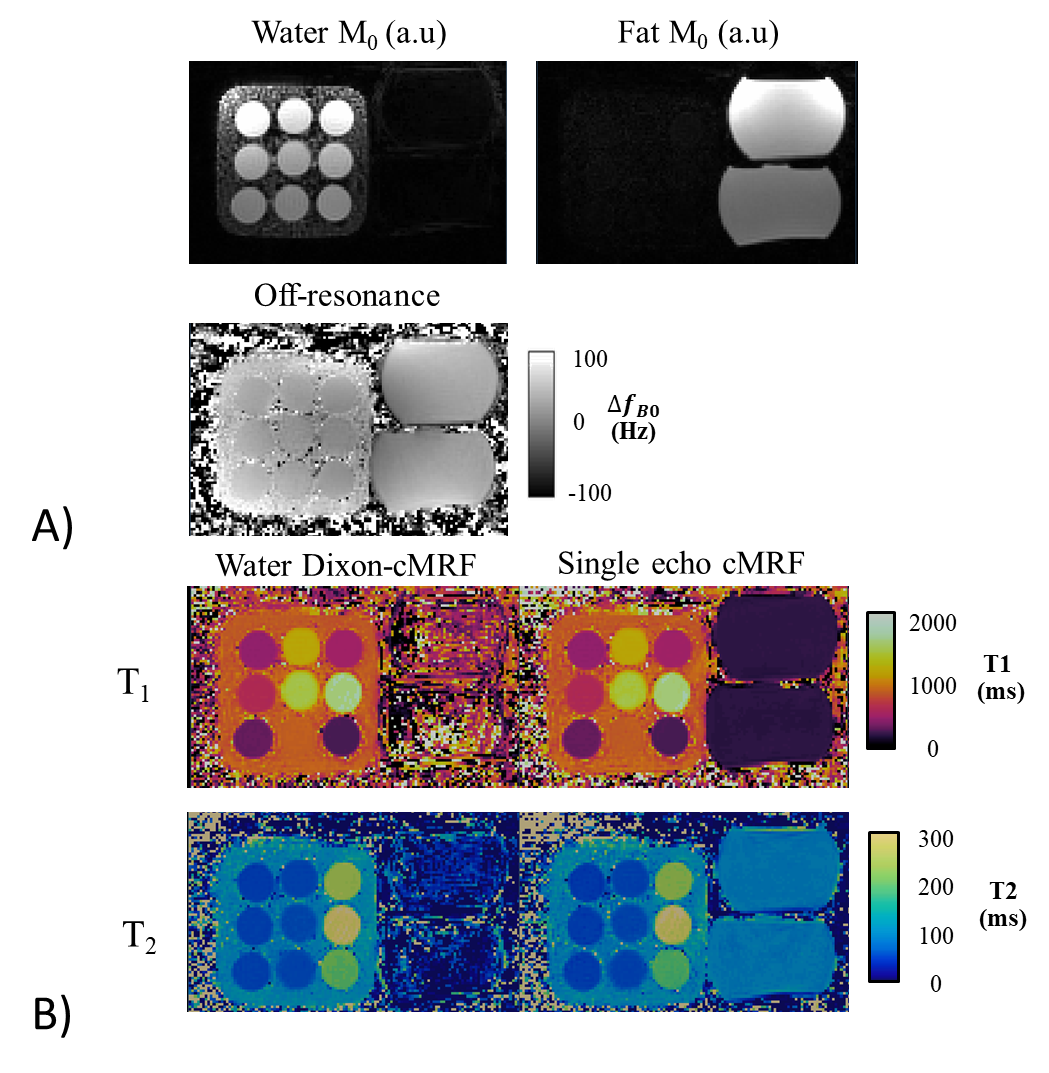


Supporting Information Figure S3. Dixon-cMRF T_1_/T_2_ phantom experiment. The standardized T1mes phantom was acquired together with two bottles of oil. A) Water M_0_, fat M_0_ and B_0_ maps showing successful separation of water and fat signals. B) Dixon-cMRF water specific and single echo cMRF (echo 1) T_1_ and T_2_ maps. As the fat signal is well suppressed the water maps match to noise in the fat bottles.

**Supporting Information Figure S4**


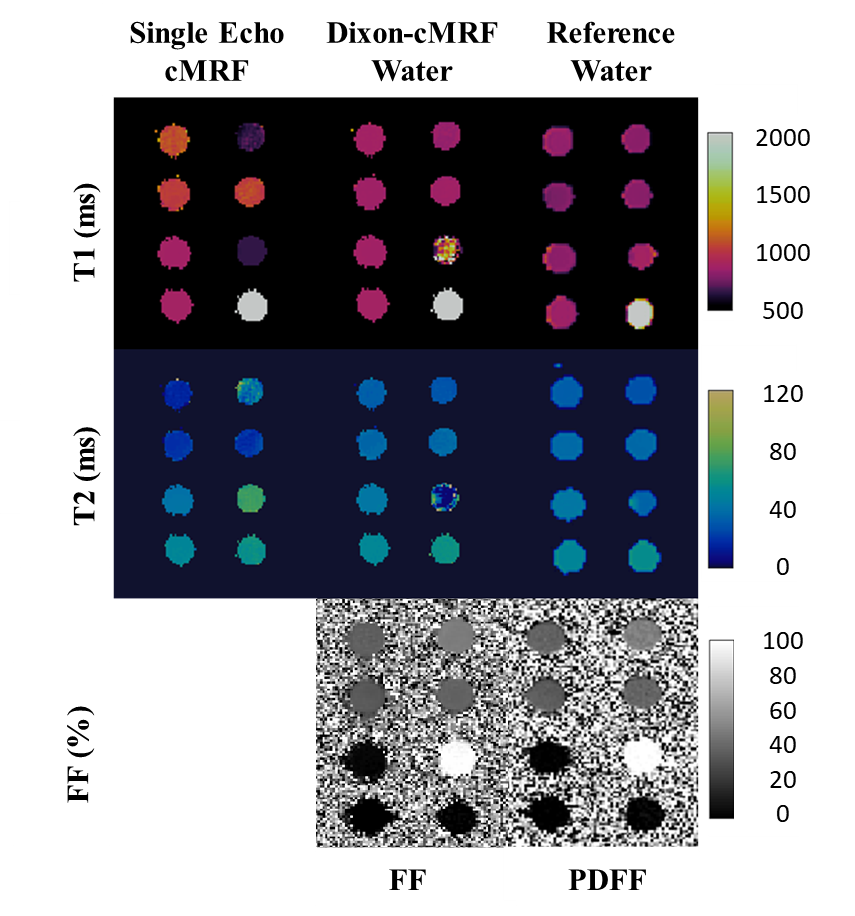


Supporting Information Figure S4. Water/fat partial volume phantom T_1_, T_2_ and FF measurements. Dixon-cMRF maps show good qualitative correspondence with reference water selective IRSE, water selective MESE and 6 echo PDFF scan while single echo cMRF (echo 1) measurements seems to map inconsistently in vials affected by water/fat partial volume as also shown in Figure 3 and Supporting Information Figure S5.B.

**Supporting Information Figure S5**


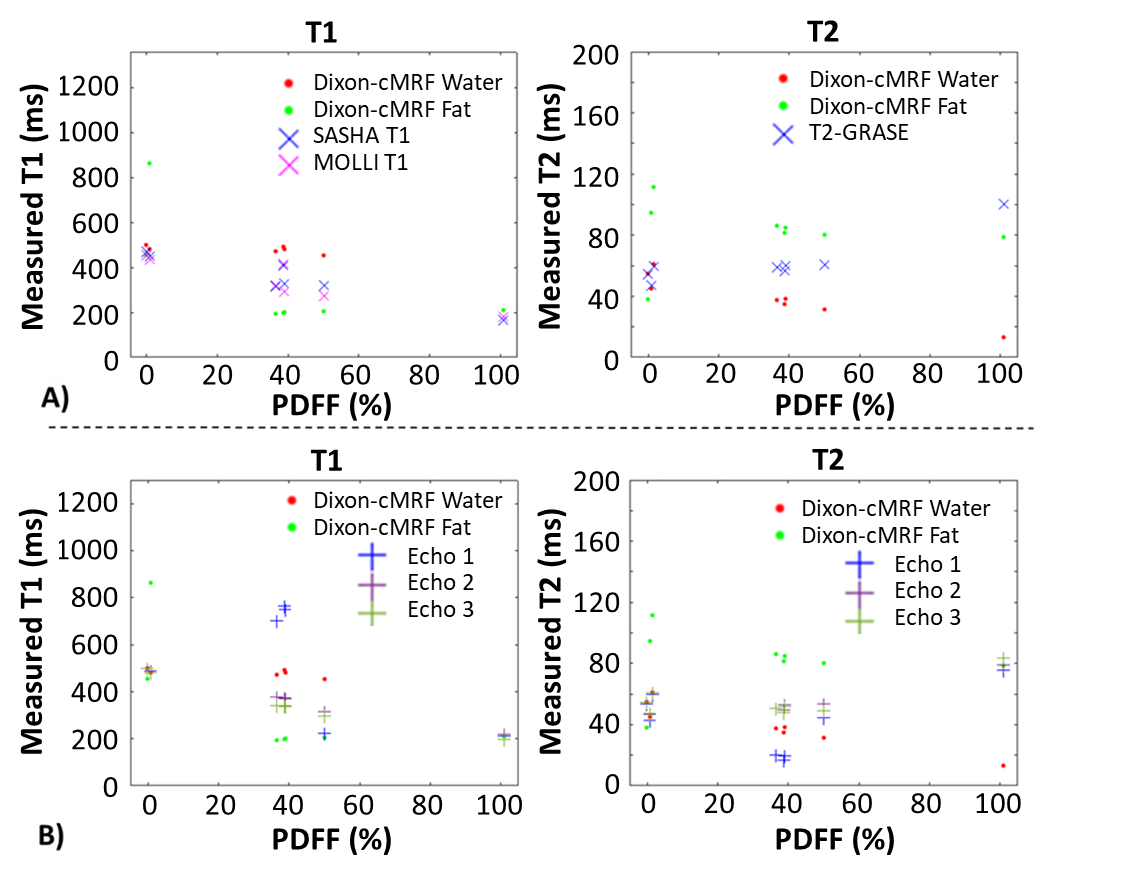


Supporting Information Figure S5. Water/fat partial volume phantom experiment. A) Comparison of water and fat specific Dixon-cMRF T_1_ to conventional SASHA and MOLLI (left), and water and fat specific Dixon-cMRF T_2_ to T_2_-GRASE (right). Conventional methods are unable to accurately estimate T_1_ or T_2_ for either of the 2 (water and fat) compartments. B) Comparison of water and fat specific Dixon-cMRF and single echo cMRF for each of the three independent echo measurements. T_1_ and T_2_ measurements in the presence of partial volume varies depending on the echo time due to different contributions of fat and water. In particular, echo 1 which is closest to out-of-phase, provides particularly poor matches in the presence of water/fat partial volume.

**Supporting Information Figure S6**


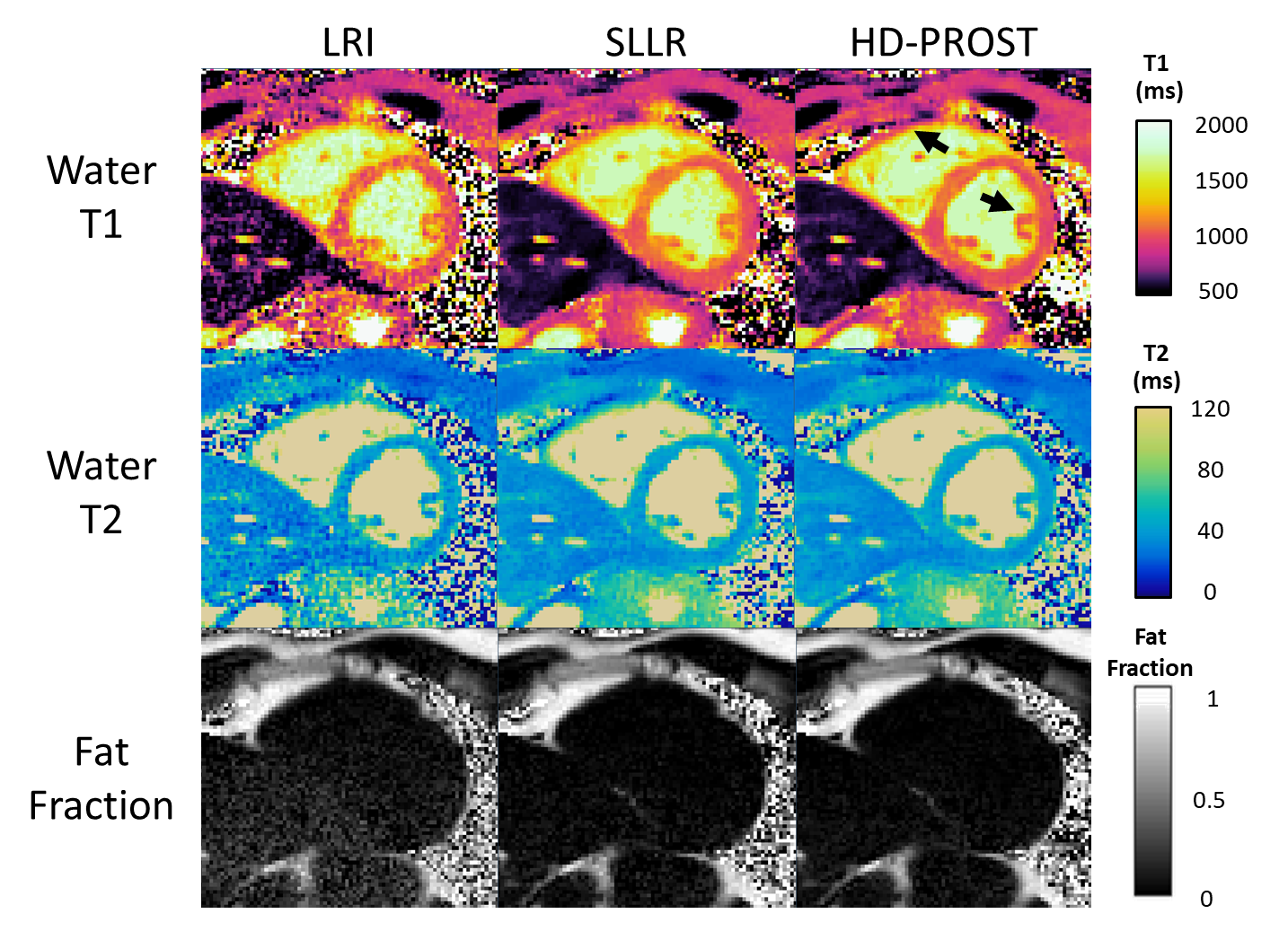


Supporting Information Figure S6. Comparison of non-regularized LRI reconstruction (left), regularized using locally low rank and Wavelet priors (SLLR) (45) (middle) and reconstructed using HD-PROST (using high order low rank regularization). LRI shows remaining noise-like artefacts which can be removed using SLLR and HD-PROST. While both provide good quality results, HD-PROST maps seem slightly sharper as shown by the black arrows.

**Supporting Information Figure S7**


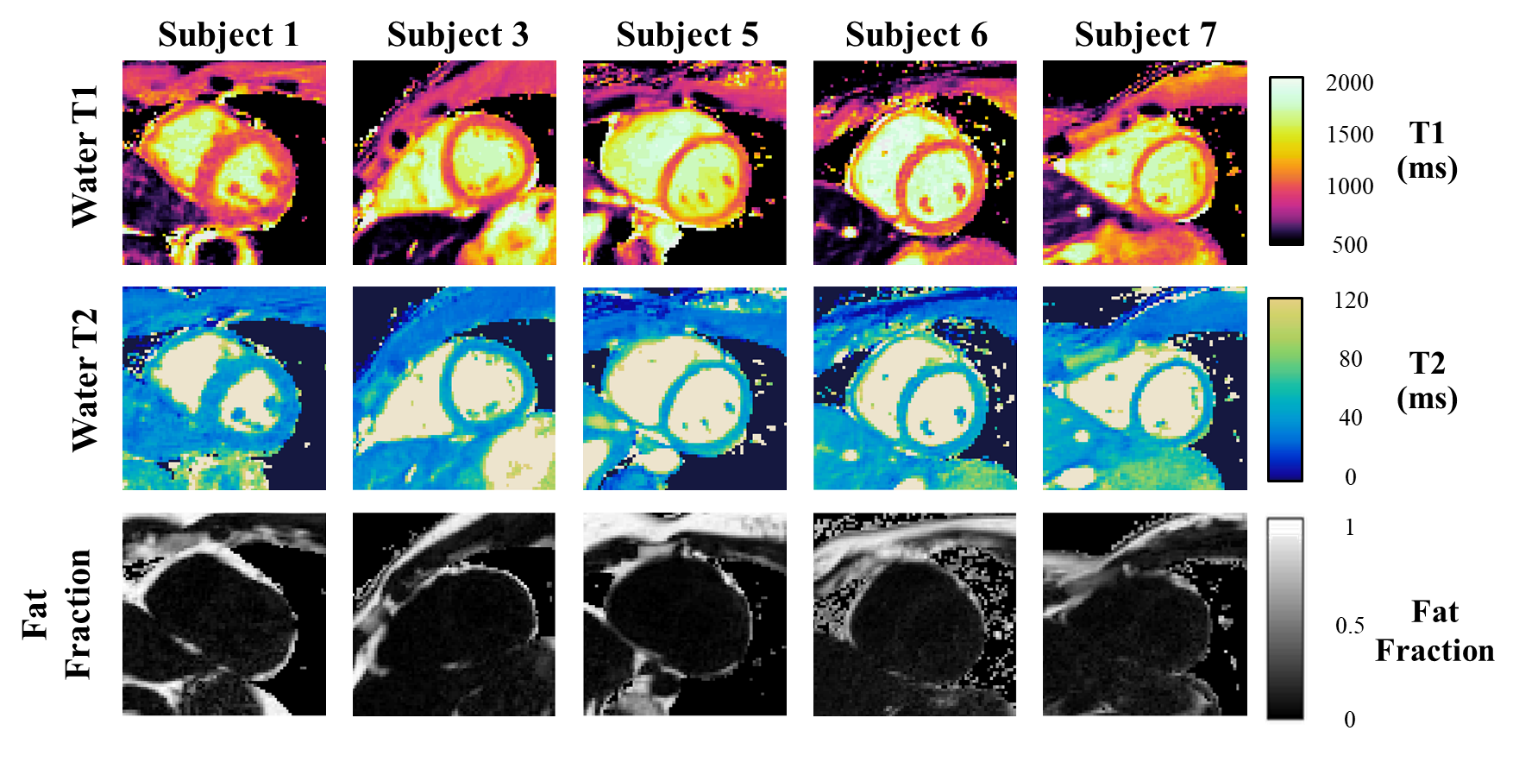


Supporting Information Figure S7. In-vivo Dixon-cMRF water T_1_, water T_2_ and fat fraction maps for five additional healthy subjects. High quality (score superior or equal to 3) water T_1_ and T_2_ maps were consistently obtained for all subjects.

**Supporting Information Figure S8**


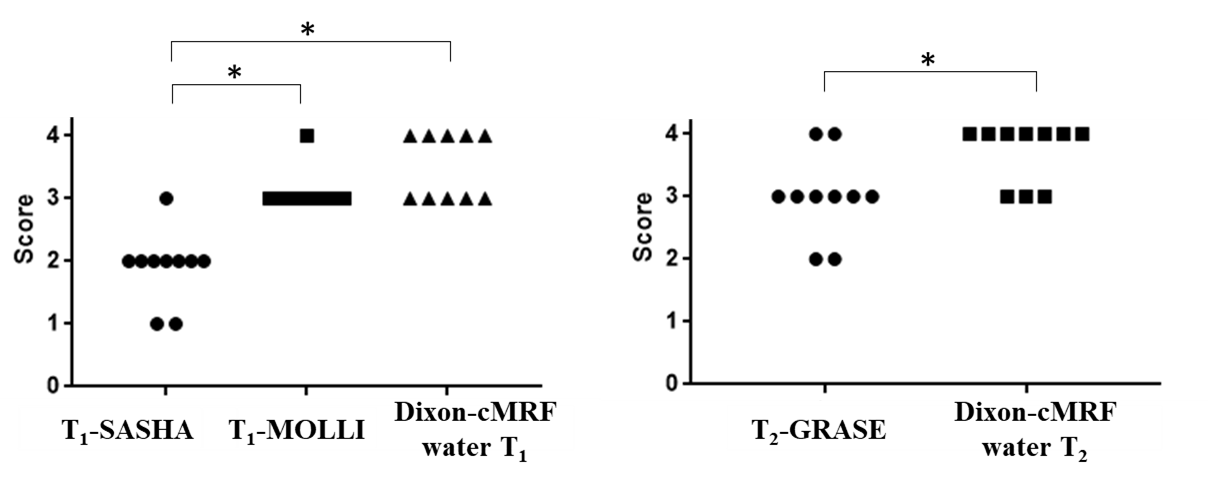


Supporting Information Figure S8. Map quality evaluation of T_1_ (left) and T_2_ (right) mapping techniques according to a 4-point scale (1- uninterpretable maps to 4- excellent map quality). Reported median scores are 2, 3 and 3.5 for SASHA, MOLLI and Dixon-cMRF water T_1_ respectively, and 3 and 4 for T_2_-GRASE and Dixon-cMRF water T_2_ respectively. All Dixon-cMRF water maps were of acceptable or excellent quality (score superior or equal to 3) and obtained equal or higher scores than their conventional counterpart SASHA, MOLLI and T_2_-GRASE maps in this study. Image scores of the proposed method were significantly better when compared to SASHA (*p<0.025) and T2-GRASE (*p<0.05). The difference in scores with MOLLI was not statistically significant.

**Supporting Information Table S1**


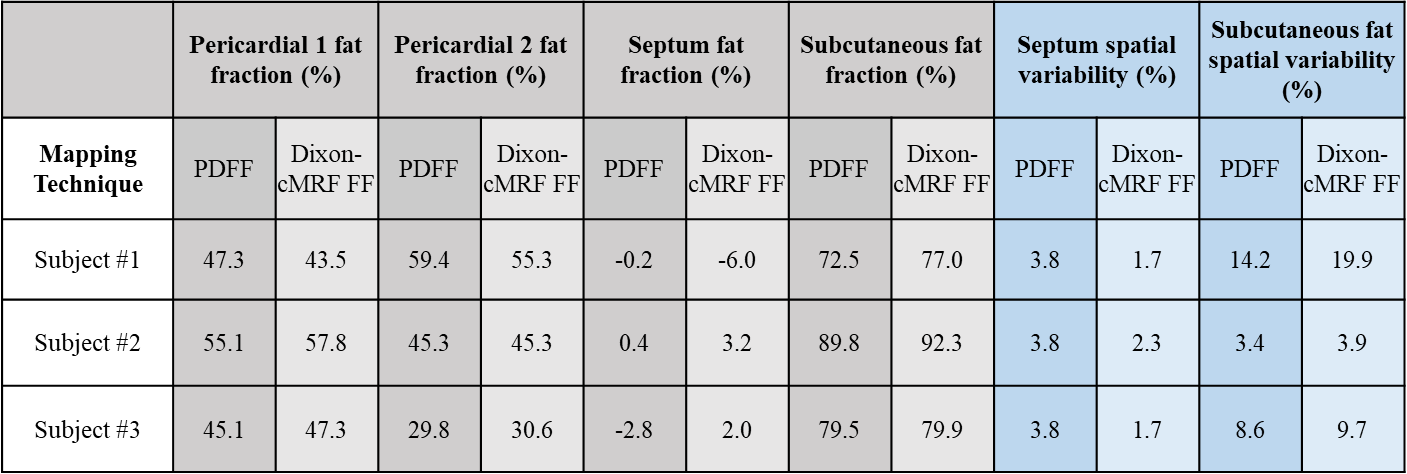


Supporting Information Table S1. Mean proton density fat fraction (PDFF) and Dixon-cMRF fat fraction (FF) measured in two separate pericardial (pericardial 1, pericardial 2), septum and subcutaneous fat ROIs in 3 healthy subjects (corresponding to the values plotted in Supporting Information Figure S9). Spatial variability of the measurement in the septum and subcutaneous fat, where homogeneous regions are expected, are also reported as a surrogate for precision. Pericardial ROIs were chosen in heterogeneous regions with varying water/fat partial volume and therefore spatial variability would not be indicative of precision and thus not reported here. Good agreement (R^2^=0.9885) was observed between the two methods and maximum absolute difference was measured at 5.8%.

**Supporting Information Figure S9**


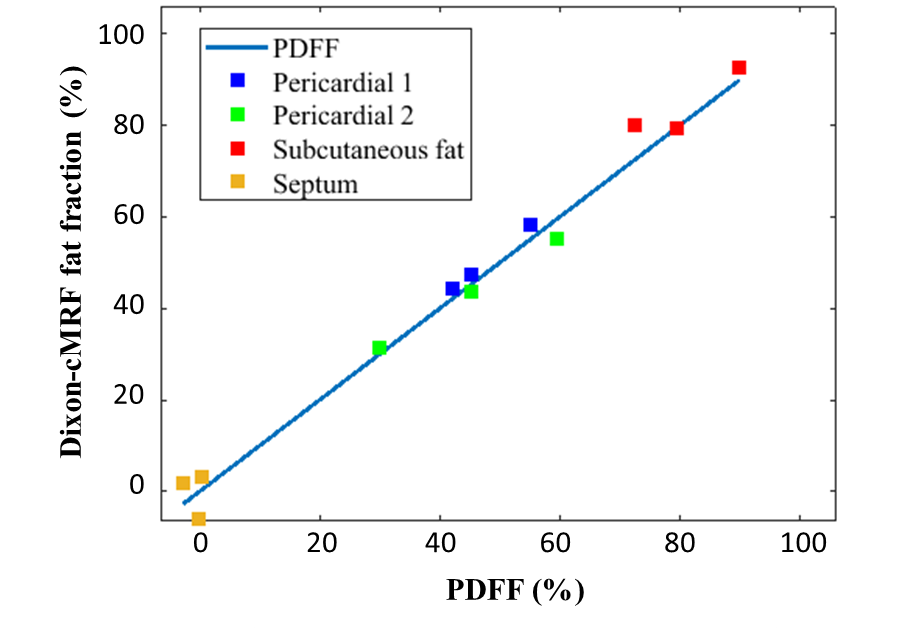


Supporting Information Figure S9. Comparison between Dixon-cMRF fat fraction and proton density fat fraction measured in four ROIs (two separate pericardial regions, subcutaneous fat and septum) for three healthy subjects. High determination coefficient R^2^=0.9885 was measured.
